# *O*-GlcNAcylation homeostasis controlled by calcium influx channels regulates multiple myeloma dissemination

**DOI:** 10.1186/s13046-021-01876-z

**Published:** 2021-03-16

**Authors:** Parinya Samart, Sudjit Luanpitpong, Yon Rojanasakul, Surapol Issaragrisil

**Affiliations:** 1 Graduate Program in Immunology, Department of Immunology, Faculty of Medicine Siriraj Hospital, Mahidol University, Bangkok, Thailand; 2 Siriraj Center of Excellence for Stem Cell Research, Faculty of Medicine Siriraj Hospital, Mahidol University, 2 Siriraj Hospital, Bangkoknoi, Bangkok, 10700 Thailand; 3 WVU Cancer Institute and Department of Pharmaceutical Sciences, West Virginia University, Morgantown, WV USA; 4 Division of Hematology, Department of Medicine, Faculty of Medicine Siriraj Hospital, Mahidol University, Bangkok, Thailand; 5 Bangkok Hematology Center, Wattanosoth Hospital, BDMS Center of Excellence for Cancer, Bangkok, Thailand

**Keywords:** *O*-GlcNAcylation, Calcium influx, Multiple myeloma, TRPM7, ORAI1, STIM1, Migration, Invasion, Dissemination

## Abstract

**Background:**

Multiple myeloma (MM) cell motility is a critical step during MM dissemination throughout the body, but how it is regulated remains largely unknown. As hypercalcemia is an important clinical feature of MM, high calcium (Ca^2+^) and altered Ca^2+^ signaling could be a key contributing factor to the pathological process.

**Methods:**

Bioinformatics analyses were employed to assess the clinical significance of Ca^2+^ influx channels in clinical specimens of smoldering and symptomatic MM. Functional and regulatory roles of influx channels and downstream signaling in MM cell migration and invasion were conducted and experimental MM dissemination was examined in a xenograft mouse model using in vivo live imaging and engraftment analysis.

**Results:**

Inhibition of TRPM7, ORAI1, and STIM1 influx channels, which are highly expressed in MM patients, and subsequent blockage of Ca^2+^ influx by CRISPR/Cas9 and small molecule inhibitors, effectively inhibit MM cell migration and invasion, and attenuate the experimental MM dissemination. Mechanistic studies reveal a nutrient sensor *O*-GlcNAcylation as a downstream regulator of Ca^2+^ influx that specifically targets cell adhesion molecules. Hyper*-O*-GlcNAcylation following the inhibition of Ca^2+^ influx channels induces integrin α4 and integrin β7 downregulation via ubiquitin-proteasomal degradation and represses the aggressive MM phenotype.

**Conclusions:**

Our findings unveil a novel regulatory mechanism of MM cell motility via Ca^2+^ influx/*O*-GlcNAcylation axis that directly targets integrin α4 and integrin β7, providing mechanistic insights into the pathogenesis and progression of MM and demonstrating potential predictive biomarkers and therapeutic targets for advanced MM.

**Supplementary Information:**

The online version contains supplementary material available at 10.1186/s13046-021-01876-z.

## Background

Multiple myeloma (MM) is a prototypical clonal B-cell malignancy characterized by an excessive growth and proliferation of terminally differentiated plasma cells (PCs) in the bone marrow (BM). Recent evidence suggests that MM arises from the BM and disseminates throughout the body. Through a process that is similar to metastasis of solid tumors, the disseminated MM cells can settle at different sites including the liver, lung, brain, and other soft tissues [1–3]. Advanced MM may be found in up to 30% of patients and is strongly correlated with poor prognosis with an overall survival of less than 6 months [4, 5]. It is widely accepted that BM microenvironment provides support for MM cell growth and survival as well as for the acquisition of aggressive phenotypes. Understanding how MM cells interact and respond to changes in the microenvironment is therefore crucial to the design of more effective anticancer therapies for MM.

One of the critical steps during MM dissemination is increased cell migration and invasion. Such increase is necessary for the intravasation of MM cells from the BM into nearby blood vessels, and for their subsequent extravasation into distant tissues [6, 7]. During the early stage of MM, these cells are exposed to a high level of Ca^2+^ in the BM niche due to increased bone resorption by the osteoclasts and consequently Ca^2+^ overload [8, 9]. Clinically, a high level of Ca^2+^ is generally regarded as the most frequent metabolic complication in MM patients [10]. Physiologically, Ca^2+^ is a ubiquitous signaling molecule regulating various cellular processes, including cell proliferation, cell death and cell motility. However, the roles of Ca^2+^ signaling, and specifically Ca^2+^ influx channels, in cell motility regulation and dissemination of MM are not well understood.

The transient receptor potential melastatin-subfamily member 7 (TRPM7) and the store-operated calcium (SOC) channels comprising of calcium release-activated calcium channel protein 1 (ORAI1) and stromal interaction molecule 1 (STIM1) are the major pathways of Ca^2+^ entry and intracellular signaling in both cancer and most excitable cells [11–13]. TRPM7 is a non-selective cation channel with notable permeability to Ca^2+^ and Mg^2+^, though it specifically facilitates Ca^2+^ influx in various cancer cells [14–16]. Additionally, TRPM7 has been shown to regulate B cell development and antigen recognition [17, 18], suggesting its role in MM pathogenesis. ORAI1 is located in the plasma membrane and regulates Ca^2+^ entry in collaboration with the endoplasmic reticulum Ca^2+^ sensor STIM1, which has been implicated in the metastasis of several solid tumors, including breast, cervical, and kidney [19–21]. Using bioinformatics database, we observed that *TRPM7*, *ORAI1* and *STIM1* mRNA expression are upregulated in patient-derived MM cells as compared to normal plasma cells (NPCs). We therefore further investigated the functional roles of these Ca^2+^ influx channels in MM cell migration and invasion and in the dissemination of MM cells in a mouse model.

An aberrant metabolism is an established hallmark of cancer [22, 23], and *O*-GlcNAcylation, a post-translational modification (PTM) in the hexosamine biosynthesis pathway, is a critical sensor of metabolic changes. We observed that cellular *O*-GlcNAcylation is dependent on changes in Ca^2+^ influx, regardless of channel type. In this study, we aimed to address the following: (a) whether *O*-GlcNAcylation plays a role in MM cell motility and dissemination; (b) whether Ca^2+^ influx regulates MM cell motility via *O*-GlcNAcylation, and (c) what are the downstream targets of Ca^2+^ influx/*O*-GlcNAcylation and the associated regulatory mechanisms.

## Materials and methods

### Reagents

2-APB, SKF96365, and thiamet G were obtained from Tocris Bioscience (Bristol, UK). PugNAc and antibodies for TRPM7, ORAI1, STIM1, OGA, *O*-GlcNAc and ITGB7 were obtained from Abcam (Cambridge, UK). Antibody for ubiquitin was from Santa Cruz Biotechnology (Dallas, TX, USA) and secondary antibodies were from EMD Millipore (Berlington, MA, USA). MG-132 and all other antibodies were from Cell Signaling Technology (Beverly, MA, USA). Other reagents were from Sigma-Aldrich (Dallas, MA, USA).

### Cells and culture

Human MM-derived cell lines RPMI8226 and National Cancer Institute (NCI)-H929 were obtained from the American Type Culture Collection (Manassas, VA, USA). Cell lines were cultured in RPMI1640 medium supplemented with 10% fetal bovine serum (FBS) and 1% penicillin-streptomycin (Invitrogen, Waltham, MA, USA) at 37 °C and 5% CO_2_. Mycoplasma contamination was regularly checked using a commercial test kit (MycoAlert™ PLUS, Lonza, Cologne, Germany) and was found to be absent in all cell lines tested.

### Oncomine™ bioinformatics database

mRNA expression analysis of clinical samples was conducted using Oncomine™ public database (https://www.oncomine.org/resource/main.html). Analyzed datasets included Zhan myeloma [24], Zhan myeloma 3 [25], and Agnelli myeloma 3 [26].

### Calcium assay

Intracellular Ca^2+^ measurements were performed using Fura-2 AM (Abcam, Cambridge, UK) as described previously with minor modifications [21]. After specific treatments, 5 × 10^4^ cells were suspended in HBSS buffer containing 4 μM Fura-2 AM and loaded onto a 96-well black plate for 1 h at 37 °C. Following an internal Ca^2+^ depletion by 2 μM thapsigargin for 10 min, 2 μM extracellular Ca^2+^ was added and Fura-2 AM signals were measured using a fluorescence microplate reader (Synergy H1, BioTek, Winooski, VT, USA) at 340/510 or 380/510 nm.

### CRISPR/Cas9-mediated gene knockdown

All-in-one pLentiCRISPR v2 plasmids carrying a single guide RNA (sgRNA) against specific target gene, spCas9 and puromycin resistance were obtained from GenScript (Piscataway, NJ, USA), while LentiCas9-blasticidin and TRPM7 sgRNA plasmids were a kind gift from Profs. Zhang, Doench, and Root (Addgene #52962 and #76111). The oligos sequences of all sgRNAs were listed in Additional file 1: Supplementary Table S1. Lentiviral particles were generated in HEK293T cells using pCMV.dR8.2 dvpr packaging and pCMV-VSV-G envelope vectors (Addgene #8454 and 8455). Cells were transduced with the virus in the presence of hexadimethrine bromide (8 μg/mL), selected with puromycin (8 μg/mL) or blasticindin (10 μg/mL), and assessed for gene knockdown efficiency by Western blotting.

### Overexpression plasmid and transfection

Cells were transfected with ITGA4 or ITGB7 plasmid (Genscript) using Lipofectamine 3000 Transfection Kit (Life Technologies, Carlsbad, CA). The transfected cells were allowed to recover for 48 h and protein level was determined by Western blotting before each experiment.

### Cell motility assay

Cell migration was assessed in a 24-well plate Transwell system (Corning, Kennebunk, ME, USA) using a cell culture insert with a pore size of 5.0 μm, while cell invasion was assessed using an insert coated with 0.5 mg/mL Matrigel (BD Bioscience, San Jose, CA, USA) [27, 28]. After starvation for 4 h, cells (1 × 10^5^ cells/well) were loaded onto the insert chamber containing low-serum (1% FBS) medium and complete medium was added to the lower chamber as a chemoattractant. Migrating/invading cells in the lower chamber were collected at 24–48 h, stained with Hoechst 33342, and visualized under an inverted fluorescence microscope (Eclipse Ti-U, Nikon, Tokyo, Japan).

### In vivo disseminated MM xenograft model

All animal studies were performed in accordance with the protocol approved by Institutional Animal Care and Use Committee of West Virginia University (WVU) (#1702005551). Cells were labeled with UBC-RFP-T2A-Luciferase dual reporter for live cell tracking. Male NSG (NOD.Cg-Prkdc^scid^. Il2rg^tm1Wjl^/SzJ) mice (WVU Transgenic Animal Core Facility, Morgantown, WV, USA) at 6–8 weeks of age were intravenously injected with 1.5 × 10^7^ luciferase-labeled cells and tumor burden was monitored on an IVIS Lumina II in Vivo Imaging system (PerkinElmer, Waltham, MA, USA). Mice were euthanized 4 weeks after the infusion or as recommended by a veterinarian. Organs were collected and imaged ex vivo to evaluate a disseminated lesion. After which, isolated organs were formalin fixed, paraffin embedded, and cut into 5-μm sections. Engraftment was confirmed by immunohistochemistry (IHC) using CD138 antibody (Invitrogen, #36–2900).

### Western blotting and immunoprecipitation (IP)

Protein was extracted using commercial protein lysis buffer (Cell Signaling Technology) supplemented with a protease inhibitor cocktail (Roche Diagnostics, Mannheim, Germany). A total protein of 30–50 μg was subjected to SDS-PAGE and transferred to PVDF membranes. Membranes were blocked with 5% fat-free milk and incubated with indicated primary antibodies overnight at 4 °C and with secondary antibodies conjugated with HRP for 2 h at room temperature. The immunoreactive proteins were captured and analyzed with ECL reagent (Millipore, Billerica, MA) using a digital imaging system (ImageQuant LAS, GE Healthcare, Pittsburgh, PA). For IP, cell lysates (100–200 μg) were incubated with anti-integrin α4 and or anti-integrin β7 antibody at 4 °C overnight, followed by incubation on ice with agarose beads for another 2 h to precipitated the protein-antibody complex. After extensive washing with ice-cold IP lysis buffer, samples were resuspended in protein loading buffer, boiled at 95 °C for 5 min, and subjected to immunoblotting.

### Statistical analysis

Data are presented as mean ± SD from three or more independent experiments. Unpaired, two-tailed Student *t*-test or one-way ANOVA with Tukey’s multiple comparison tests were used to determine statistical significance at the significance level of *P* < 0.05 (GraphPad Prism, San Diego, CA, USA).

## Results

### *TRPM7*, *ORAI1* and *STIM1* levels are upregulated in MM

**Fig. 1:**
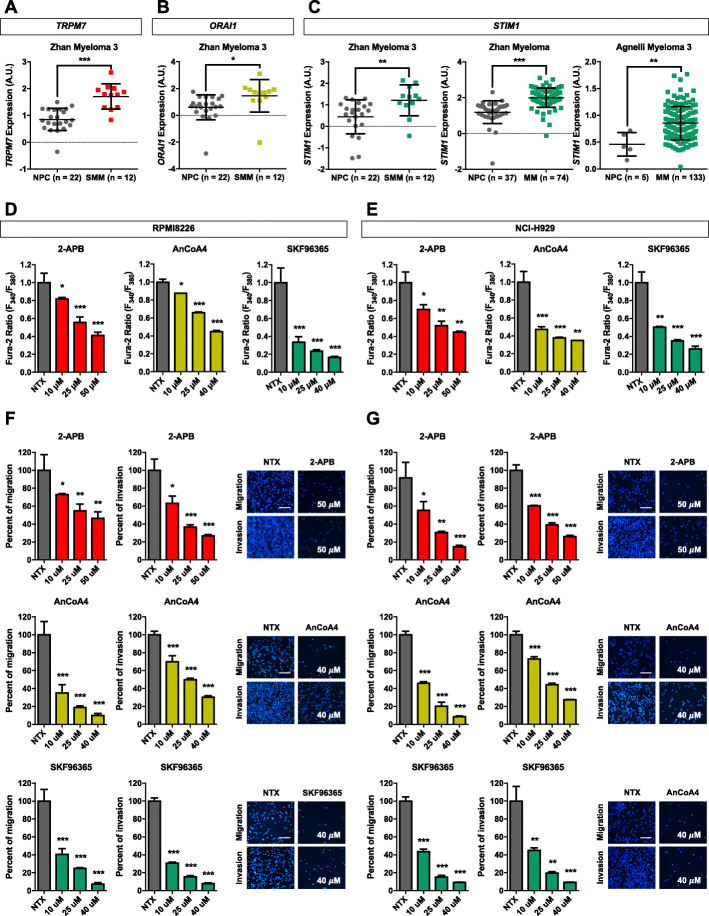
Upregulation of *TRPM7*, *ORAI1* and *STIM1* in clinical MM specimens and their inhibition by small molecule inhibitors in human MM cells. **a**-**c** Differential mRNA expression of *TRPM7*, *ORAI1* and *STIM1* in SMM and/or MM tissues in comparison to NPC in clinical datasets available on Oncomine™ bioinformatics database. *P < 0.05, **P < 0.01, ***P < 0.0001 versus NPC; two-tailed Student’s *t*-test. **d, e** Effect of SMIs of TRPM7, ORAI1, and STIM1 on intracellular Ca^2+^ levels. Human MM-derived RPMI8226 and NCI-H929 cells were treated with different concentrations of 2-APB (0–50 μM), AnCoA4 (0–40 μM) and SKF96365 (0–40 μM), and free intracellular Ca^2+^ levels were measured by a fluorescence plate reader using Fura-2 AM as a probe. Fura-2 signals were measured after a depletion of internal Ca^2+^ stores at 340/510 and 380/510 nm and reported as relative Fura-2 ratio to non-treated cells (NTX). **f, g** RPMI8226 and NCI-H929 cells were similarly treated with 2-APB (0–50 μM) (upper), AnCoA4 (0–40 μM) (middle), and SKF96365 (0–40 μM) (lower), and cell migration and invasion were evaluated by Transwell assays at 48 h. The penetrating cells were stained by Hoechst 33342 dye, quantified, and reported as percentage to NTX cells. Representative micrographs of migrating/invading cells stained with Hoechst 33342 are shown (see also Additional file 2: Fig. S4 for additional micrographs). Scale bar = 100 μm. Data are mean ± SD (*n* = 4). *P < 0.05, **P < 0.01, ***P < 0.001 versus NTX; two-tailed Student’s *t*-test

mRNA expression analysis of critical Ca^2+^ influx channels was first performed in human clinical specimens available on Oncomine™ bioinformatics database. We observed that *TRPM7*, *ORAI1*, and *STIM1* expression was significantly higher in smoldering MM (SMM), a precancerous form of MM, when compared to NPCs (Fig. 1A–C). An upregulation of *STIM1* expression in symptomatic MM was observed in two other datasets (Fig. 1C), while information on *TRPM7* and *ORAI1* expression in symptomatic MM was not available. These data suggest that aberrant Ca^2+^ influx channels may be involved in MM pathogenesis and progression and support the previous study demonstrating a close relation between elevated SOC influx channels and clinical outcome of MM patients [29].

### Inhibition of Ca^2+^ influx channels suppresses MM cell migration and invasion

To investigate the potential role of TRPM7, ORAI1, and STIM1 in MM cell motility, we first used various small-molecule inhibitors (SMIs) of the channels, including 2-APB, AnCoA4, and SKF96365, as schematically depicted in Additional file 2: Fig. S1. To ensure that their effect on cell motility was not due to their adverse effects on cell viability and proliferation, we used sub-cytotoxic concentrations of the SMIs, i.e. up to 50 μM for 2-APB and up to 40 μM for AnCoA4 and SKF96365 in MM RPMI8226 and NCI-H929 cells (see Additional file 2: Fig. S2 for MTT assay and Fig. S3 for cell cycle analysis and Annexin V-PE/7-AAD apoptosis assay) in our study. Free intracellular Ca^2+^ in response to 2-APB treatment was evaluated using Fura-2 AM as a fluorescent probe. Figure 1D and E shows that 2-APB, AnCoA4, and SKF96365 dose-dependently inhibited Ca^2+^ influx in both RPMI8226 and NCI-H929 cells, indicating that Ca^2+^ influx channels are functional in these MM cells and that they are responsive to the SMI treatments. Phenotypically, we observed that the cells treated with 2-APB, AnCoA4, and SKF96365 were less migratory and invasive by 30–95% depending on the dose, as evaluated by Transwell assays (Fig. 1F and G; Additional file 2: Fig. S4). These data suggest that Ca^2+^ influx channels, particularly TRPM7, ORAI1, and STIM1, are critical for MM cell migration and invasion.

### Depletion of TRPM7, ORAI1, and STIM1 suppresses MM cell motility

**Fig. 2:**
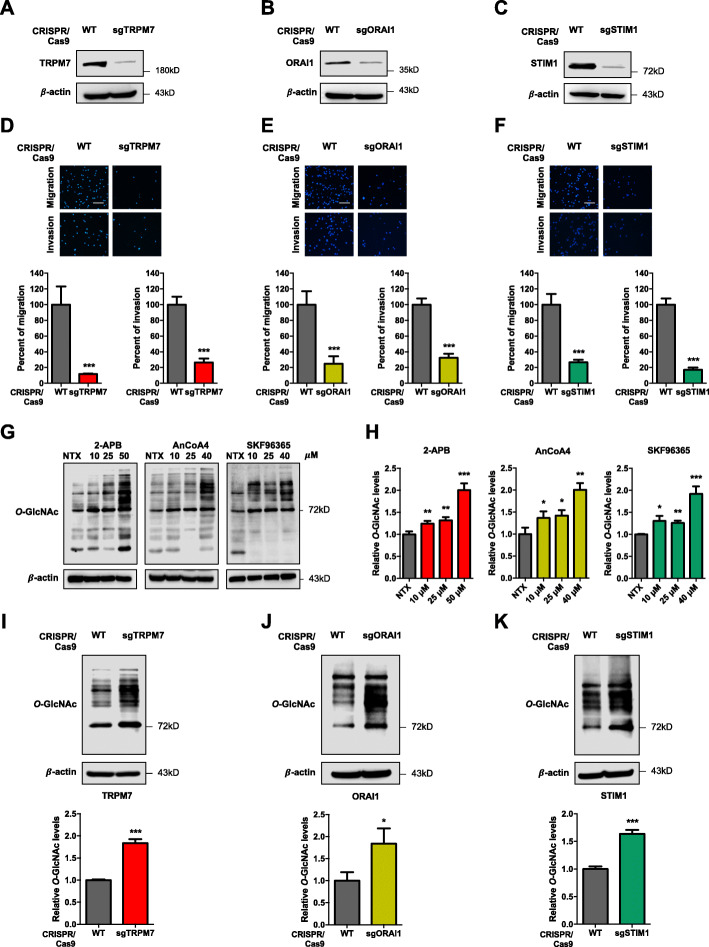
Inhibition of TRPM7, ORAI1 and STIM1 reduces cell migration and invasion in MM cells likely in concomitant with a decreased cellular *O*-GlcNAcylation. **a–c** Western blot analysis showing TRPM7, ORAI1, and STIM1 knockdown efficiency in sgTRPM7, sgORAI1 and sgSTIM1 RPMI8226 cells, respectively. **d–f** Cell migration and invasion were evaluated by Transwell assays at 48 h, where the penetrating cells were stained by Hoechst 33342 dye. (upper) Representative micrographs of migrating/invading cells are shown. Scale bar = 100 μm. (lower) Quantitative data showing the percentage of migrating/invading cells relative to WT control cells. Data mean ± SD (*n* = 4). ***P < 0.001 versus WT; two-tailed Student’s *t*-test. **g** RPMI8226 cells were treated with 2-APB (0–50 μM), AnCoA4 (0–40 μM), and SKF96365 (0–40 μM) for 24 h, and cellular *O*-GlcNAcylation level was evaluated by Western blotting. **h** Quantitative analysis of *O*-GlcNAcylation level in treated cells, normalized to β-actin and relative to non-treated cells (NTX). Data are mean ± SD (n = 4). *P < 0.05, **P < 0.01, ***P < 0.001 versus NTX; two-tailed Student’s *t*-test. **i–k** Cellular *O*-GlcNAcylation level was compared between sgTRPM7, sgORAI1 or sgSTIM1 and WT RPMI8226 cells. Data are mean ± SD (n = 4). *P < 0.05, ***P < 0.001 versus NTX; two-tailed Student’s *t*-test

To specifically determine the role of TRPM7, ORAI1, and STIM1 influx channels in MM cell motility, CRISPR/Cas9 system was used to repress the specific gene expression in MM cells. The cells were transduced with lentiviral particles comprising sgRNA against *TRPM7* (sgTRPM7), *ORAI1* (sgORAI1), and *STIM1* (sgSTIM1) and Cas9, and their effect on protein expression was determined by Western blotting (Fig. 2A–C; Additional file 2: Fig. S5). Figure 2D–F shows that sgTRPM7, sgORAI1, and sgSTIM1-transduced cells exhibited significantly lower migratory and invasive activities than vector control (wild-type, WT) cells. We also verified that the sgTRPM7, sgORAI1 and sgSTIM1 cells exhibited a similar proliferative activity, cell cycle profile, and apoptosis rate as their respective WT control cells (Additional file 2: Fig. S6), thus ruling out their anti-proliferative effect on the observed cell motility. Together, the results from our CRISPR/Cas9 gene depletion and SMI studies strongly indicate that TRPM7, ORAI1, and STIM1 are key regulators of MM cell motility.

### Ca^2+^ influx acts upstream of cellular *O*-GlcNAcylation

Recent studies have demonstrated a plausible crosstalk between Ca^2+^ influx and cellular *O*-GlcNAcylation in excitable cells [30]. To test this possibility in MM cells, cellular *O*-GlcNAc level was manipulated in MM RPMI8226 cells and its effect on Ca^2+^ influx channels was determined by Western blotting, and vice versa. Additional file 2: Fig. S7 shows that the levels of TRPM7, ORAI1, and STIM1 were relatively unchanged in cells treated with OGA inhibitor PugNAc (10–50 μM) or thiamet G (2.5–10 μM), which caused an increase in *O*-GlcNAc level. In contrast, cells treated with Ca^2+^ influx channel inhibitors, including 2-APB (10–50 μM), AnCoA4 (10–40 μM), and SKF96365 (10–40 μM), exhibited a dose-dependent increase in cellular *O*-GlcNAc level as compared to non-treated control (Fig. 2G and H). The increase in cellular *O*-GlcNAc level upon inhibition of TRPM7, ORAI1, and STIM1 was validated in sgTRPM7, sgORAI1, and sgSTIM1 cells, which displayed elevated *O*-GlcNAc levels as compared to WT cells (Fig. 2I–K). These data indicate that *O*-GlcNAcylation is regulated by Ca^2+^ influx via TRPM7, ORAI1, and STIM1 channels and that *O*-GlcNAcylation may regulate MM cell motility.

### Elevated *O*-GlcNAcylation reduces migration and invasion of MM cells

**Fig. 3:**
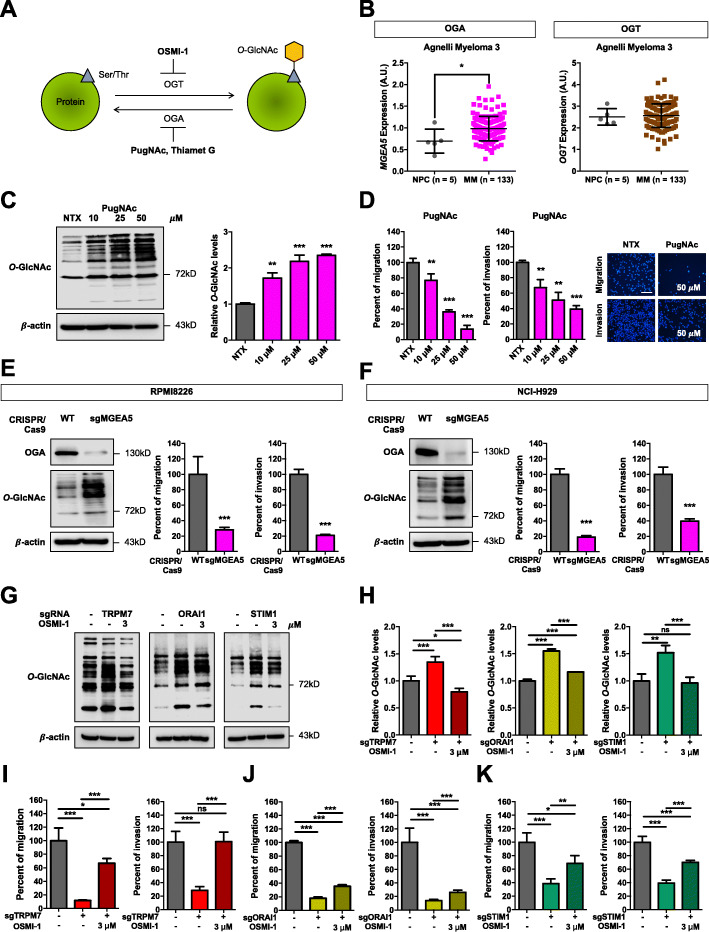
MM cell motility is regulated by Ca^2+^ influx/*O*-GlcNAc axis. **a** Schematic diagram showing *O*-GlcNAcylation on protein’s serine/threonine residues and SMIs that modulate OGT, including OSMI-1, and OGA, including PugNAc and thiamet G. **b** *MGEA5* (encoding OGA) and *OGT* mRNA expression in MM tissues in comparison to NPC in a clinical dataset available on Oncomine™ bioinformatics database. Data mean ± SD. *P < 0.05 versus NPC; two-tailed Student’s *t*-test. **c** Human MM-derived RPMI8226 cells were treated with various concentrations of PugNAc (0–50 μM) for 24 h and cellular O-GlcNAcylation was analyzed by Western blotting to confirm its induction. (right) Quantitative analysis of *O*-GlcNAcylation, normalized to β-actin and relative to the non-treated cells (NTX). Data are mean ± SD (*n* = 4). **P < 0.01, ***P < 0.001 versus NTX; two-tailed Student’s *t*-test. **d** Cell migration and invasion in response to PugNAc (0–50 μM) were evaluated by Transwell assays at 48 h, where the penetrating cells were stained with Hoechst 33342 dye. Scale bar = 100 μm. Data are mean ± SD (n = 4). *P < 0.05, **P < 0.01, ***P < 0.001 versus NTX; two-tailed Student’s *t*-test. **e, f** RPMI8226 and NCI-H929 cells were genetically knocked down with sgRNA sequence against *MGEA5* (encoding OGA) in the CRISPR/Cas9 system. Western blot analysis showing OGA knockdown efficiency in sgMGEA5 cells. After which, cell migration and invasion were evaluated by Transwell assays at 48 h. (see also Additional file 2: Fig. S10C and D for representative micrographs of migrating/invading cells). Quantitative data showing the percentage of migrating/invading cells relative to WT control cells. Data mean ± SD (n = 4). ***P < 0.001 versus WT; two-tailed Student’s *t*-test. **g–k** Rescue experiments were performed in sgTRPM7, sgORAI1, and sgSTIM1 RPMI8226 cells by treating the cells with OGT inhibitor OSMI-1. **g, h** Cellular O-GlcNAcylation level in sgTRPM7, sgORAI1, and sgSTIM1 cells in the presence or absence of OSMI-1 (3 μM) for 24 h was evaluated by Western blotting. Representative immunoblots **(g)** and quantitative analysis of *O*-GlcNAcylation levels **(h)** indicate that OSMI-1 could reverse the hyper-*O*-GlcNAcylation in sgTRPM7, sgORAI1, and sgSTIM1 cells. Data mean ± SD (*n* = 4). *P < 0.05, **P < 0.01, ***P < 0.001 versus non-treated WT control cells or non-treated sgTRPM7, sgORAI1 or sgSTIM1 cells; one-way ANOVA with Tukey’s multiple comparison test. ns, not significant. **i–k** Cell migration and invasion in sgTRPM7, sgORAI1, and sgSTIM1 cells in the presence or absence of OSMI-1 (3 μM) were evaluated by Transwell assays at 48 h. (see also Additional file 2: Fig. S14A–C for representative micrographs of migrating/invading cells). Quantitative data showing the percentage of migrating/invading cells relative to non-treated WT cells. Data mean ± SD (n = 4). *P < 0.05, **P < 0.01, ***P < 0.001 versus non-treated WT control cells or non-treated sgTRPM7, sgORAI1 or sgSTIM1 cells; one-way ANOVA with Tukey’s multiple comparison test. ns, not significant

Although *O*-GlcNAcylation has been associated with metastasis of various solid tumors [31], its role in MM cell migration and invasion has not been demonstrated. In this study, we used OGT and OGA inhibitors, as schematically depicted in Fig. 3A, to evaluate the dose-response effect of *O*-GlcNAcylation on cell motility. To ascertain the clinical significance of *O*-GlcNAcylation in MM, we also analyzed *OGT* and *MGEA5* (encoding OGA) expression in MM patients using the Oncomine™ database. We found that *MGEA5* expression was significantly higher in MM patients as compared to NPCs, whereas the expression of *OGT* was not significantly different in the two groups (Fig. 3B). These results suggest a reduced *O*-GlcNAcylation in MM. Treatment of MM cells with PugNAc (10–50 μM) and thiamet G (2.5–10 μM) decreased cell migration and invasion in a dose-dependent manner as compared to non-treated control, in an inverse relationship with the *O*-GlcNAc level (Fig. 3C and D; Additional file 2: Fig. S8 and S9). Consistently, genetic inhibition of *MGEA5* by CRISPR/Cas9 (sgMGEA5), which caused an increase in *O*-GlcNAcylation, reduced the migration and invasion of MM cells (Fig. 3E and F; Additional file 2: Fig. S10). It should be noted that inhibition of OGA by both SMIs and CRISPR/Cas9 did not affect cell proliferation, cell cycle or apoptosis (Additional file 2: Fig. S11 and S12), indicating that the observed inhibitory effects were indeed due to impaired cell motility. Similar results were obtained in human MM-derived NCI-H929 cells (Additional file 2: Fig. S13). These findings are in good agreement with the TRPM7, ORAI1, and STIM1 inhibition data showing their effect on hyper-*O*-GlcNAcylation, and with the clinical data linking hypo-*O*-GlcNAcylation with aggressive MM.

### Hypo-*O*-GlcNAcylation rescues impaired cell motility upon inhibition of TRPM7, ORAI1, and STIM1

Based on the findings that inhibition of TRPM7, ORAI1, and STIM1 elevated cellular *O*-GlcNAcylation level, and that inhibition of such channels and *O*-GlcNAcylation reduced MM cell migration and invasion, we postulated that TRPM7, ORAI1, and STIM1 may regulate MM cell motility through *O*-GlcNAcylation. Hence, hypo-*O*-GlcNAcylation should be able to rescue the effects on cell motility caused by inhibition of the channels. To test this possibility, sgTRPM7, sgORAI1, and sgSTIM1 cells were treated with OGT inhibitor OSMI-1 (3 μM), which reduced *O*-GlcNAcylation (Fig. 3G and H), and its effects on cell migration and invasion were determined by Transwell assays. Figure 3I–K shows that OSMI-1 rescued the inhibitory effects in sgTRPM7, sgORAI1, and sgSTIM1 cells, substantiating that TRPM7, ORAI1, and STIM1 regulate MM cell motility through *O*-GlcNAcylation (Additional file 2: Fig. S14). These results demonstrated a novel role of Ca^2+^ influx/*O*-GlcNAc via TRPM7, ORAI1, and STIM1 channels in regulating MM cell motility.

### Disruption of Ca^2+^ influx/*O*-GlcNAc axis suppresses tumor burden in MM xenograft model

**Fig. 4:**
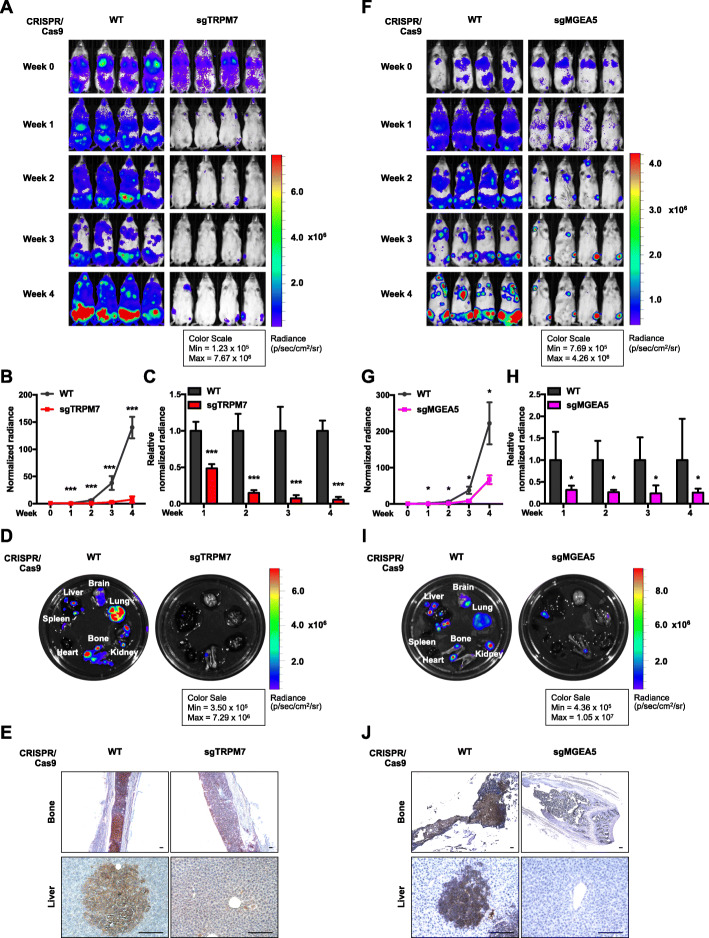
Inhibition of Ca^2+^ influx channels and hyper-*O*-GlcNAcylation impairs MM dissemination in vivo. **a–e** Luciferase-labeled sgTRPM7 or WT RPMI8226 cells were injected into NSG mice via tail vein. **A** Representative bioluminescence imaging of mice taken at the time of inoculation (week 0) and weekly post-injection (week 1, 2, 3 and 4). **b, c** Quantification of whole-body luminescence signals normalized to their initial signal at week 0 (**b**) and relative to WT control cells (**c**). Data mean ± SD (*n* = 5). ***P < 0.001 versus WT cells; two-tailed Student’s *t*-test. **d** Representative ex vivo imaging from isolated internal organs including brain, lung, kidney, bone, heart, spleen, and liver at week 4 post-injection (see also Additional file 2: Fig. S15 for quantification of ex vivo IVIS images). **e** Representative IHC micrographs of femur bone and liver sections staining for human CD138 (see also Additional file 2: Fig. S17 for additional IHC micrographs). Scale bar = 100 μm. **f–j** Luciferase-labeled sgMGEA5 or WT RPMI8226 cells were similarly injected into NSG mice via tail vein and bioluminescence imaging and IHC staining were similarly performed as described in A–E (see also Additional file 2: Fig. S18 for additional IHC micrographs)

Having demonstrated that Ca^2+^ influx acts upstream of *O*-GlcNAcylation in regulating MM cell motility, we experimentally verified the involvement of TRPM7, which showed the most pronounced effect on cell motility, and *O*-GlcNAcylation in MM dissemination in a xenograft mouse model [32, 33]. sgTRPM7, sgMGEA5 and WT cells were genetically labeled with luciferase and intravenously injected into NSG mice via tail vein to induce systemic disease, followed by a weekly bioluminescence imaging for quantitative analysis of tumor progression. Bioluminescence analyses revealed that both sgTRPM7 and sgMGEA5 cells yielded substantially lower whole-body signals at 1, 2, 3 and 4 weeks post-injection comparing to WT cells (Fig. 4A, B, F, and G), suggesting less tumor burden and experimental dissemination of MM cells. For better comparison, the luminescence signals in each group were normalized to their initial signals at week 0 and relative to WT control. Figure 4C and H confirmed a lower myeloma burden in mice bearing sgTRPM7 and sgMGEA5 cells at week 1 to 4 post-injection. To support the finding of MM dissemination, mice were euthanatized at the end of the experiments and various organs, including brain, lung, kidney, bone, heart, spleen, and liver, were collected for ex vivo bioluminescence imaging and histochemistry. Figure 4D and I demonstrated substantially lower tumor signals in the various organs in mice bearing sgTRPM7 and sgMGEA5 cells as compared to those in WT mice. These results are consistent with the IHC finding showing a decreased number of infiltrated MM cells in the various organs, including bone (femur) and liver, and the flow cytometric analysis of human CD138+ cells in the BM of femur bone (Fig. 4E and J; see also Additional file 2: Fig. S15–18). Together, these results support the in vitro findings that Ca^2+^ influx and hypo-*O*-GlcNAcylation are critical regulators of MM cell motility and dissemination.

### Elevated *O*-GlcNAcylation decreases MM cell motility via suppression of integrin α4 and integrin β7

**Fig. 5:**
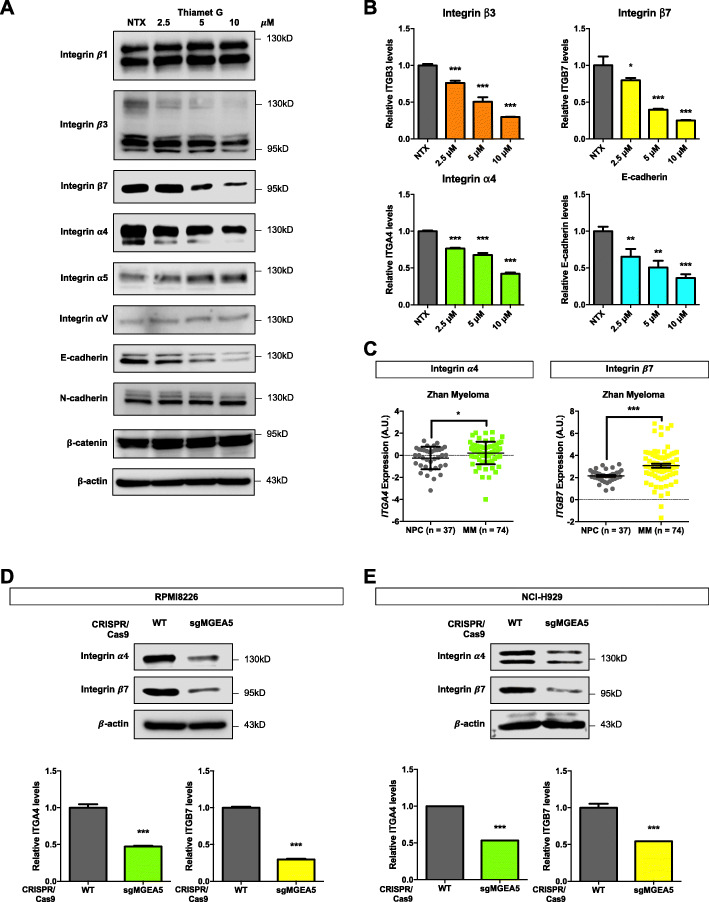
Hyper-*O*-GlcNAcylation suppresses MM cell motility via ITGA4 and ITGB7. **a** Human MM-derived RPMI8226 cells were treated with various concentrations of thiamet G (0–10 μM) and analyzed for cell adhesion proteins, including ITGB1, ITGB3, ITGB7, ITGA4, ITGA5, ITGAV, E-cad, N-cad, and B-cat by Western blotting. **b** Quantitative analysis of the indicated proteins, normalized to β-actin and relative to the non-treated cells (NTX) (see also Additional file 2: Fig. S19 for an additional quantification). Data mean ± SD (*n* = 4). *P < 0.05, **P < 0.01, ***P < 0.001 versus NTX; two-tailed Student’s *t*-test. **c** *ITGA4* and *ITGB7* expression in MM tissues in comparison to NPC in a clinical dataset available on Oncomine™ bioinformatics database. Data mean ± SD. *P < 0.05, ***P < 0.001 versus NPC; two-tailed Student’s *t*-test. **d, e** OGA-knockdown sgMGEA5 RPMI8226 and NCI-H929 cells were evaluated for ITGA4 and ITGB7 levels by Western blotting to confirm that they are downstream of *O*-GlcNAcylation. (lower) Quantitative analysis of ITGA4 and ITGB7 levels, normalized to β-actin and relative to WT control cells. Data mean ± SD. ***P < 0.001 versus WT; two-tailed Student’s *t*-test

**Fig. 6:**
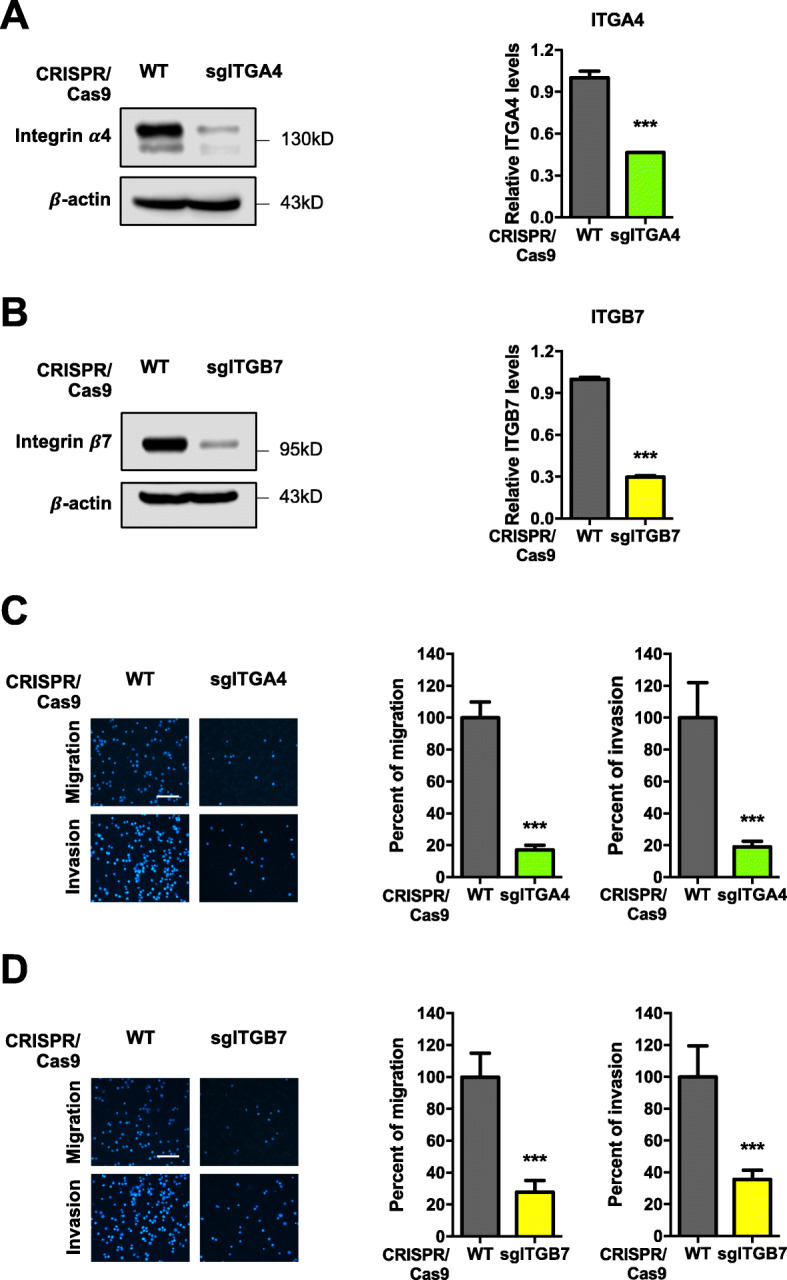
Inhibition of ITGA4 and ITGB7 critically suppresses MM cell motility. **a**, **b** Human MM-derived RPMI8226 cells were genetically knocked down with sgRNA against *ITGA4* and *ITGB7* by CRISPR/Cas9. (left) Western blot analysis showing knockdown efficiency in sgITGA4 (**a**) and sgITGB7 (**b**) cells. **c, d** Cell migration and invasion were evaluated in sgITGA4 (**c**) and sgITGB7 (**d**) cells by Transwell assays at 48 h, where the penetrating cells were stained with Hoechst 33342 dye. (left) Representative micrographs of migrating/invading cells are shown. Scale bar = 100 μm. (right) Quantitative data showing the percentage of migrating/invading cells relative to WT control cells. Data mean ± SD (n = 4). ***P < 0.001 versus WT; two-tailed Student’s *t*-test

*O*-GlcNAcylation has been long known to modify proteins and alter their functions and cellular pathways [34]. To gain a more in-depth understanding of the underlying mechanisms, we used a literature mining approach to identify cell adhesion molecules that are involved in MM progression, including the integrin family of proteins such as integrin β1 (ITGB1), β3 (ITGB3), β7 (ITGB7), α4 (ITGA4), α5 (ITGA5) and αV (ITGAV), E-cadherin (E-cad), N-cadherin (N-cad), and β-catenin (B-cat) [35–38]. The expression level of these proteins in response to thiamet G (2.5–10 μM) treatment was examined in MM cells by Western blotting. Figure 5A and B shows that thiamet G minimally affected ITGB1, ITGAV, ITGA5, N-cad, and B-cat expression, but significantly downregulated ITGB3, ITGA4, ITGB7, and E-cad in a dose-dependent manner (see also Additional file 2: Fig. S19A). Bioinformatics analysis of the adhesion molecules in MM patients using Oncomine™ database revealed that *ITGA4* and *ITGB7* were significantly upregulated in MM tissues as compared to NPCs, while *ITGB3* and *CDH1* expression was not different (Fig. 5C; see also Additional file 2: Fig. S19B), suggesting the role of ITGA4 and ITGB7 in MM cell adhesion. Genetic inhibition of *ITGA4* and *ITGB7* by CRISPR/Cas9 in MM cells, designated as sgITGA4 and sgITGB7 cells (Fig. 6A and B), also showed a substantial reduction in MM cell migration and invasion when compared to WT control (Fig. 6C and D), while having minimal effect on cell proliferation, cell cycle and apoptosis (Additional file 2: Fig. S20). The change in ITGA4 and ITGB7 expression following *O*-GlcNAcylation was verified in sgMGEA5 cells (Fig. 5 D and E), thus confirming that ITGA4 and ITGB7 are key regulators of MM cell motility under *O*-GlcNAcylation.

**Fig. 7:**
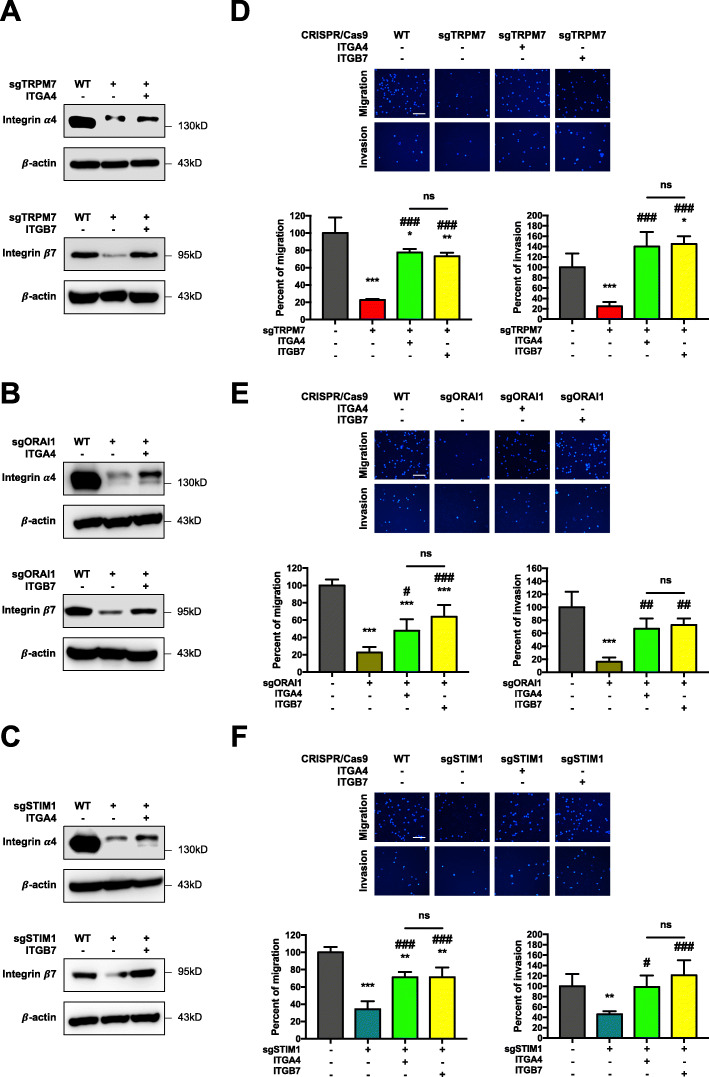
ITGA4 and ITGB7 are favorable downstream targets of Ca^2+^ influx/*O*-GlcNAc-mediated MM cell motility. **a–c** Rescue experiments were performed in sgTRPM7 (**a**), sgORAI1 (**b**), and sgSTIM1 (**c**) RPMI8226 cells by transfection of the cells with ITGA4, ITGB7 or control pcDNA plasmid. ITGA4 (upper) and ITGB7 (lower) levels were evaluated by Western blot analysis at 48 h post-transfection prior to experiments and representative blots were shown. **d–f** Cell migration and invasion in ITGA4 or ITGB7-overexpressing sgTRPM7 (**d**), sgORAI1 (**e**), and sgSTIM1 (**f**) cells by Transwell assays at 48 h, where the penetrating cells were stained with Hoechst 33342 dye. Quantitative data showing the percentage of migrating/invading cells relative to transfected control cells. Data mean ± SD (*n* = 3). *P < 0.05, **P < 0.01, ***P < 0.001 versus pcDNA-transfected control cells; one-way ANOVA with Tukey’s multiple comparison test. ^#^*P* < 0.05, ^##^*P* < 0.01, ^###^*P* < 0.001 versus pcDNA-transfected sgTRPM7, sgORAI1, or sgSTIM1 cells; one-way ANOVA with Tukey’s multiple comparison test. ns, not significant

To further validate that ITGA4 and ITGB7 are downstream of Ca^2+^ influx under the Ca^2+^ influx/*O*-GlcNAc axis, rescue experiments were conducted in which ITGA4 and ITGB7 plasmid were ectopically overexpressed in various Ca^2+^ influx-inhibited cells, including sgTRPM7, sgORAI1, and sgSTIM1 RPMI8226 cells, and their effects on cell motility and protein level were determined. Figure 7A–C showed the reduced ITGA4 and ITGB7 levels in sgTRPM7, sgORAI1, and sgSTIM1 cells, indicating that Ca^2+^ influx via TRPM7, ORAI1, and STIM1 channels regulated ITGA4 and ITGB7 abundance. Overexpression of ITGA4 and ITGB7 did restore the reduced migration and invasion in sgTRPM7, sgORAI1, and sgSTIM1 cells (Fig. 7D–F), in agreement with the increased ITGA4 and ITGB7 levels, strengthening that the ITGA4 and ITGB7 levels and their functional contributions were indeed regulated by the Ca^2+^ influx/*O*-GlcNAc axis.

Notably, we also monitored the pan matrix metalloproteinase (MMP) activity using MMP Activity Fluorometric Assay Kit (Abcam) in sgTRPM7, sgORAI1, sgSTIM1 cells as well as in sgMGEA5 cells and WT control cells since MMPs are known to be involved in cancer cell motility of various cancers and have been reported to promote MM dissemination [39, 40]. However, our results showed no significant difference in total MMP activity in sgTRPM7, sgORAI1, sgSTIM1, and sgMGEA5 cells, indicating that MMPs may not play a significant role in the MM dissemination under the experimental Ca^2+^ influx/*O*-GlcNAc conditions (Additional file 2: Fig. S21).

### *O*-GlcNAcylation of ITGA4 and ITGB7 induces ubiquitin-mediated proteasomal degradation

**Fig. 8:**
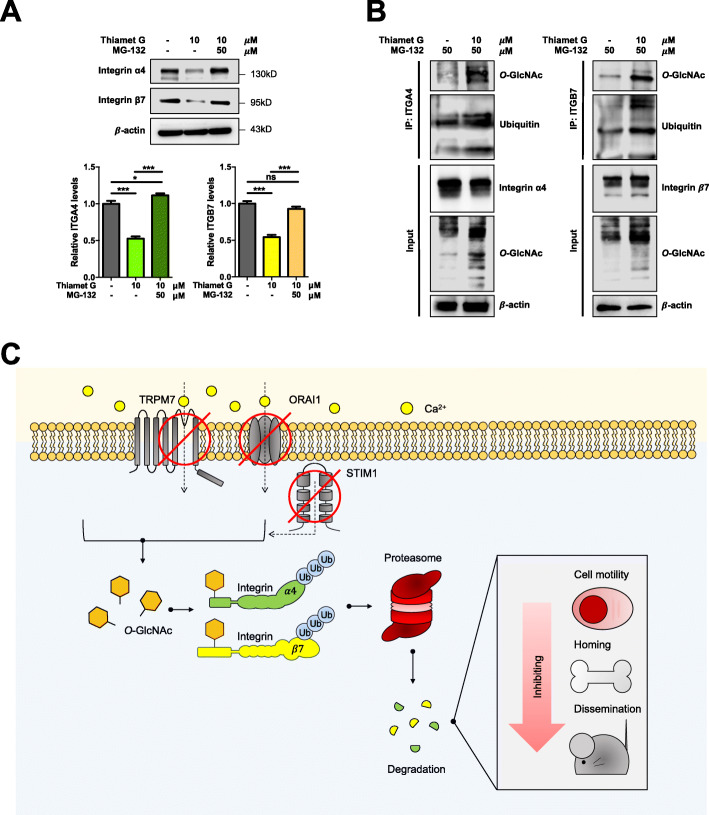
Hyper-*O*-GlcNAcylation promotes ubiquitin-mediated proteasomal degradation of ITGA4 and ITGB7. **a** RPMI8226 cells were pretreated with the proteasome inhibitor MG-132 (50 μM) for 3 h, followed by thiamet G (10 μM) treatment for 6 h, and ITGA4 and ITGB7 levels were analyzed by Western blotting. (lower) Quantitative analysis of ITGA4 (left) and ITGB7 (right) levels, normalized to β-actin and relative to non-treated cells (NTX). Data mean ± SD (n = 4). *P < 0.05, ***P < 0.001 versus NTX cells or thiamet G-treated cells alone; one-way ANOVA with Tukey’s multiple comparison test. ns, not significant. **b** RPMI8226 cells were pretreated with MG-132 to prevent proteasomal degradation and then treated with thiamet G (10 μM) for 6 h. The cells were then subjected to immunoprecipitation (IP) with anti-ITGA4 and anti-ITGB7 antibodies, and the immune complexes were detected for *O*-GlcNAcylation or ubiquitination by Western blotting. IP inputs were also evaluated for ITGA4, ITGB7 or β-actin for comparison. **c** A schematic illustration for the regulation of MM cell motility and dissemination by Ca^2+^ influx/*O*-GlcNAc axis. Ca^2+^ influx channels, including TRPM7, ORAI1, and STIM1, regulate Ca^2+^ entry in MM cells. Following Ca^2+^ influx channels inhibition, cellular *O*-GlcNAcylation is elevated and activates ITGA4 and ITGB7. *O*-GlcNAcylation of ITGA4 and ITGB7 induces downregulation of the two adhesion proteins via ubiquitin (Ub)-proteasomal degradation, causing a repression of MM cell motility and dissemination

Proteasomal degradation is known to play a major role in cellular proteostasis via the regulation of protein stability and degradation [41]. We tested whether *O*-GlcNAcylation regulates ITGA4 and ITGB7 stability by proteasomal degradation by first performing a rescue experiment using an established proteasome inhibitor, MG-132 (50 μM). Figure 8A shows that ITGA4 and ITGB7 downregulation by thiamet G was rescued by MG-132 treatment, indicating that *O*-GlcNAcylation regulates ITGA4 and ITGB7 via a proteasome-dependent pathway. Since *O*-GlcNAcylation and ubiquitination are PTMs that similarly occur at Ser/Thr residues [42], we next determined the interplay between the two on ITGA4 and ITGB7 proteins using co-immunoprecipitation. Figure 8B reveals physical interaction between ITGA4 and ITGB7 and the association between *O*-GlcNAcylation and ubiquitination of the two proteins. Elevated ITGA4 and ITGB7 *O*-GlcNAcylation by thiamet G caused an increase in ITGA4 and ITGB7 ubiquitination. These results indicate that *O*-GlcNAcylation of ITGA4 and ITGB7 promotes their ubiquitin-proteasomal degradation, leading to reduced protein expression and attenuated MM cell motility.

## Discussion

This study unveils a novel mechanism of MM cell motility and dissemination regulation via Ca^2+^ influx and *O*-GlcNAcylation axis. Depletion of Ca^2+^ influx channels, including TRPM7, ORAI1, and STIM1, in human-derived MM cells causes hyper-*O*-GlcNAcylation and subsequent ubiquitin-proteasome mediated degradation of ITGA4 and ITGB7, leading to their decreased expression and consequential reduction in MM cell motility and dissemination (Fig. 8C). These cell adhesion molecules as targets of Ca^2+^ influx/*O*-GlcNAcylation axis could have a major clinical implication in MM therapies since ITGA4 and ITGB7 are highly expressed in the majority of MM tissues, but not in NPCs.

Ca^2+^ is a ubiquitous second messenger that controls many essential cellular processes, such as cell proliferation, differentiation, and death [43]. The involvement of Ca^2+^ signaling in various oncogenic and metastatic processes initially arose from the observations that breast, lung, and prostate cancers often metastasize to the bone, causing osteolysis and high Ca^2+^ accumulation in the tumor ecosystem [44]. In contrast to solid tumors, BM is the primary site of MM development and dysregulated Ca^2+^ environment, i.e., due to bone resorption, and therefore could play a key role in MM dissemination. Oncomine™ bioinformatics studies showed that *TRPM7*, *ORAI1*, and *STIM1* are highly expressed in SMM and MM patients’ tissues compared with healthy NPCs. In addition, an increased Ca^2+^ influx is linked to high-grade MM (Additional file 2: Fig. S22), suggesting that the elevated Ca^2+^ influx channels may serve as prognostic markers of MM progression. To substantiate these observations, we additionally performed comparative gene expression analysis between NPCs and newly diagnosed, aggressive MM that were subsequently treated with three total therapies (TT3) using datasets available on Gene Expression Omnibus (GEO; accession number GSE5900 and GSE2658) (Additional file 2: Fig. S23) [45, 46]. Consistent with the Oncomine™ database analyses, *TRPM7* and *STIM1* are upregulated in TT3 MM cohort, though we did not observe a significant change in *ORAI1* expression. Remarkably, the direct linkage between TRPM7 expression and MM progression has never been reported, although an increased expression of ORAI1 and STIM1 in MM that has previously been described and linked to poor clinical outcomes. For example, ORAI1 and STIM1 were found to express at a high level in BM tissues of stage III MM compared to stage I/II MM [29]. Analysis of MM patients, regardless of stage, also indicated a direct relationship between STIM1 expression and the duration of progression-free survival.

While the importance of Ca^2+^ influx and its channels in cancer progression has been well recognized, the precise mechanisms of regulation are unclear and likely to depend on cancer type and cellular context. Genetic downregulation of TRPM7 was shown to inhibit hypoxia-induced cell motility in androgen-independent prostate cancer cells. Such inhibition was mediated through proteasomal degradation of HIF-1α, which resulted in stabilization and increased binding of HIF-1α to RACK1, [47] suggesting the role of Ca^2+^ influx in protein stability and function. In the present study, we demonstrated for the first time the role of Ca^2+^ influx through TRPM7, STIM1 and ORAI1 channels in controlling protein stability and function via *O*-GlcNAcylation. We provided compelling evidence that Ca^2+^ influx through the described Ca^2+^ channels acts upstream of *O*-GlcNAcylation in an inverse relationship (Fig. 2) and that such Ca^2+^ influx regulates MM cell motility through *O*-GlcNAcylation (Fig. 3).

The roles of *O*-GlcNAcylation in the pathogenesis and progression of solid tumors have been well documented. Hyper*-O*-GlcNAcylation or elevated *O*-GlcNAcylation levels has been observed in various tumors, including breast, colon, pancreas, liver, bladder, gastric and lung, and has been attributed to an upregulated OGT and/or downregulated OGA expression [31]. Hyper*-O*-GlcNAcylation was found to cause an acquired apoptosis resistance in lung carcinoma through p53 and c-Myc, independent of p53 status, and induce cell migration and invasion through caveolin-1 and c-Myc [48]. However, the involvement of *O*-GlcNAcylation in the pathogenesis of hematologic malignancies has been far less studied and may be different from that of solid tumors. In chronic lymphocytic leukemia (CLL), indolescent clinical behaviors of CLL cells and favorable clinical outcomes correlate well with the higher level of *O*-GlcNAcylation [49]. Likewise, hyper*-O*-GlcNAcylation induced by OGA inhibition was found to sensitize bortezomib-induced apoptosis and reverse bortezomib resistance in mantle cell lymphoma (MCL), suggesting the potential utility of OGA inhibitors such as ketoconazole in adjuvant therapy of MCL [50]. In myeloid malignancies, OGT directly stabilizes ASXL1 by *O*-GlcNAcylation and drives myeloid differentiation associated with the pathogenesis of myelodysplastic syndrome (MDS) [51]. *O*-GlcNAcylation may also be involved in the pathogenesis of MM since it is involved in the lymphopoiesis of B cells [52, 53], which terminally differentiate into plasma cells.

As MM cells initially reside in the BM microenvironment and adhere to extracellular matrix and/or BM stromal cells, cell adhesion molecules such as integrins and cadherins are crucial for their BM homing, cell survival, proliferation and drug resistance. Integrins are the major adhesion molecules that are highly expressed in MM cells [35]. We demonstrated in this study that ITGA4 and ITGB7 are a direct target of *O*-GlcNAcylation that regulates MM cell motility and dissemination downstream of Ca^2+^ influx signaling. Bioinformatics analyses using Oncomine™ and the GEO datasets strengthen the clinical significance of ITGA4 and ITGB7, as their gene expression tend to be upregulated in the MM tissues when compared to healthy NPCs, although *ITGB7* was not statistically significant (*p* = 0.0788) in one of the datasets (Fig. 5 and Additional file 2: Fig. S23). Hyper-*O*-GlcNAcylation causes a concomitant decrease in ITGA4 and ITGB7 expression (Fig. 5), which could be reversed by the addition of proteasome inhibitor MG-132 (Fig. 8), indicating that *O*-GlcNAcylation interferes with proteasomal degradation of ITGA4 and ITGB7. Accumulating evidence also indicates the crosstalk between *O*-GlcNAcylation and ubiquitination to promote either protein stability or turnover [42]. Hyper-*O*-GlcNAcylation of ITGA4 and ITGB7 was shown to promote their ubiquitination and subsequent proteasomal degradation. Although, hyper-*O*-GlcNAcylation was reported to induce ITGB1 activation in Hela cells via focal adhesion complex formation [54], this is the first demonstration of the regulation of ITGA4 and ITGB7 by *O*-GlcNAcylation via ubiquitin-proteasome mediated degradation pathway. It is worth noting that constitutive activation of ITGB7 has been observed in MM cells but not in non-hematopoietic cells/tissues, making ITGB7 a potential therapeutic target for chimeric antigen receptor (CAR) T cells [33].

## Conclusion

In summary, the evidence presented here demonstrated that MM cell motility and dissemination could be functionally modulated by the Ca^2+^ influx/*O*-GlcNAcylation regulatory axis that directly targets ITGA4 and ITGB7 (Fig. 8C). Our novel findings on the molecular pathways and interactions provide mechanistic insights into the pathogenesis and progression of MM, and identify potential predictive biomarkers and drug targets for advanced MM. Herein, genetic inhibition of Ca^2+^ influx channel TRPM7 and OGA, which act upstream of ITGA4 and ITGB7, effectively inhibited experimental MM dissemination in vivo, suggesting the potential clinical applications of Ca^2+^ influx and *O*-GlcNAcylation modulators. It is worth noting that TRPM7, STIM1/ORAI1, and OGA are reported to be druggable targets [55–57] and an administration of currently available SMIs in vivo, e.g. NS8593 for TRPM7, and PugNAc and thiamet G for OGA, could be used as a promising tool for preclinical assessment [58–60]. However, development of potent, selective, and high-affinity SMIs for targeting Ca^2+^ influx and/or *O*-GlcNAcylation for cancer therapeutics is challenging and still in progress. Further studies would contribute to investigate the effects of novel SMIs in the in vivo disseminated MM xenograft model, which could be advantageous for the future MM treatment to achieve long-term control of the disease.

## Supplementary Information

**Additional file 1: Supplementary Table S1.** The Oligos sequences of single guide RNA (sgRNA).

**Additional file 2: Fig. S1.** Schematic representation of small molecule inhibitors (SMIs) targeting Ca^2+^ influx channels used in this study. **Fig. S2.** Small molecule inhibition of Ca^2+^ influx channels do not affect cell viability of multiple myeloma (MM) cells. **Fig. S3.** Small molecule inhibition of Ca^2+^ influx channels do not affect cell cycle and apoptosis of MM cells. **Fig. S4.** Small molecule inhibition of Ca^2+^ influx channels dose-dependently inhibits MM cell motility. **Fig. S5.** Ca^2+^ influx channels sgRNA decreases the protein levels of TRPM7, ORAI1, and STIM1, respectively, in RPMI8226 cells. **Fig. S6.** Knockdown of TRPM7, ORAI1, and STIM1 do not affect cell viability, cell cycle, and apoptosis of human MM-derived RPMI8226 cells. **Fig. S7.** Expression of Ca^2+^ influx channels are not affected by increasing levels of *O-*GlcNAc modification. **Fig. S8.** Hyper-*O*-GlcNAcylation by PugNAc treatment inhibits MM cell motility. **Fig. S9.** Hyper-*O*-GlcNAcylation by thiamet G treatment inhibits MM cell motility. **Fig. S10.** Hyper-*O*-GlcNAcylation by sgRNA against *MGEA5* in the CRISPR/Cas9 system inhibit MM cell motility. **Fig. S11.** Hyper-*O*-GlcNAcylation by OGA inhibition does not affect cell viability, cell cycle, and apoptosis of RPMI8226 cells. **Fig. S12.** Knockdown of OGA does not affect cell viability, cell cycle, and apoptosis of human MM-derived RPMI8226 cells. **Fig. S13.** Elevated *O*-GlcNAcylation similarly reduces the migration and invasion of human MM-derived NCI-H929 cells. **Fig. S14.** *O*-GlcNAcylation acts as a down-stream of Ca^2+^ influx channels. **Fig. S15.** Inhibition of Ca^2+^ influx channels and OGA knockdown impair MM cell dissemination in vivo. **Fig. S16.** Inhibition of Ca^2+^ influx channels and OGA knockdown inhibit MM spreading to BM. **Fig. S17.** TRPM7 depletion inhibits MM cell dissemination in vivo. **Fig. S18.** OGA depletion inhibits MM cell dissemination in vivo. **Fig. S19.** Elevated *O*-GlcNAcylation does not significantly associate with integrin β1 (ITGB1), β3 (ITGB3), α5 (ITGA5) and αV (ITGAV), E-cadherin (E-cad), N-cadherin (Ncad), and β-catenin (B-cat). **Fig. S20.** Knockdown of ITGA4 and ITGB7 does not affect cell viability, proliferation and apoptosis of human MM-derived RPMI8226 cells. **Fig. S21.** Matrix metalloproteinase (MMP) activity upon an inhibition of Ca^2+^ influx channels or induction of *O*-GlcNAcylation. **Fig. S22.** Upregulation of mRNA expression of Ca^2+^ influx channels is associated with advanced MM clinical stages. **Fig. S23.** mRNA expression of Ca^2+^ influx channels, *O*-GlcNAc cycling enzymes, and integrins were analyzed using microarray data available on Gene Expression Omnibus (GEO) under accession number (GSE5900 and GSE2658).

## Data Availability

The datasets generated and/or analyzed during the current study are available from the corresponding author on reasonable request. Supplementary information is available.

## Abbreviations

B-cat: β-catenin
BM: Bone marrow
Ca^2+^: Calcium
CAR: Chimeric antigen receptor
CLL: Chronic lymphocytic leukemia
E-cad: E-cadherin
ITGA4: Integrin α4
ITGA5: Integrin α5
ITGAV: Integrin αV
ITGB1: Integrin β1
ITGB3: Integrin β3
ITGB7: Integrin β7
MCL: Mantle cell lymphoma
Mg^+^: Magnesium
MM: Multiple myeloma
N-cad: N-cadherin
NPCs: Normal plasma cells
*O*-GlcNAc: *O*-linked β-N-acetylglucosamine
ORAI1: Calcium release-activated calcium channel protein 1
PCs: Plasma cells
PTM: Post-translational modification
sgRNA: Single guide RNA
SMIs: Small-molecule inhibitors
SMM: Smoldering multiple myeloma
SOC: Store-operated calcium
STIM1: Stromal interaction molecule 1
TRPM7: Transient receptor potential melastatin-subfamily member 7
WT: Wild-type
