# Supplementary material for: O-GlcNAcylation homeostasis controlled by calcium influx channels regulates multiple myeloma dissemination
Source: J Exp Clin Cancer Res. 2021 Mar 16;40:100. doi: 10.1186/s13046-021-01876-z (PMC7968185; doi:10.1186/s13046-021-01876-z)
Supplement: Supplementary file 1 — Additional file 1: Supplementary Table S1. The Oligos sequences of single guide RNA (sgRNA). [file 13046_2021_1876_MOESM1_ESM.pdf]

Supplementary Table S1. The Oligos sequences of single guide RNA (sgRNA)

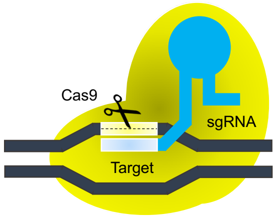

Schematic illustration of CRISPR/Cas9-mediated TRPM7, ORAI1 and STIM1 knockdown. sgRNA sequences that specifically recognize the target DNA region.

Oligo name	Oligo sequence (5'–3')
sgTRPM7	CAAATATAAGTGCCACCACA
sgORAI1	CAACGTGCACAATCTCAACT
sgSTIM1	CTCAGTATGAGGAGACCTTC
sgMGEA5	GGTGTGGATAGCAACGTAGT
sgITGA4	GTGTTTGTGTACATCAACTC
sgITGB7	GGGACGCACAAGCCTTCGAG
