# Supplementary material for: O-GlcNAcylation homeostasis controlled by calcium influx channels regulates multiple myeloma dissemination
Source: J Exp Clin Cancer Res. 2021 Mar 16;40:100. doi: 10.1186/s13046-021-01876-z (PMC7968185; doi:10.1186/s13046-021-01876-z)
Supplement: Supplementary file 2 — Additional file 2: Fig. S1. Schematic representation of small molecule inhibitors (SMIs) targeting Ca2+ influx channels used in this study. Fig. S2. Small molecule inhibition of Ca2+ influx channels do not affect cell viability of multiple myeloma (MM) cells. Fig. S3. Small molecule inhibition of Ca2+ influx channels do not affect cell cycle and apoptosis of MM cells. Fig. S4. Small molecule inhibition of Ca2+ influx channels dose-dependently inhibits MM cell motility. Fig. S5. Ca2+ influx channels sgRNA decreases the protein levels of TRPM7, ORAI1, and STIM1, respectively, in RPMI8226 cells. Fig. S6. Knockdown of TRPM7, ORAI1, and STIM1 do not affect cell viability, cell cycle, and apoptosis of human MM-derived RPMI8226 cells. Fig. S7. Expression of Ca2+ influx channels are not affected by increasing levels of O-GlcNAc modification. Fig. S8. Hyper-O-GlcNAcylation by PugNAc treatment inhibits MM cell motility. Fig. S9. Hyper-O-GlcNAcylation by thiamet G treatment inhibits MM cell motility. Fig. S10. Hyper-O-GlcNAcylation by sgRNA against MGEA5 in the CRISPR/Cas9 system inhibit MM cell motility. Fig. S11. Hyper-O-GlcNAcylation by OGA inhibition does not affect cell viability, cell cycle, and apoptosis of RPMI8226 cells. Fig. S12. Knockdown of OGA does not affect cell viability, cell cycle, and apoptosis of human MM-derived RPMI8226 cells. Fig. S13. Elevated O-GlcNAcylation similarly reduces the migration and invasion of human MM-derived NCI-H929 cells. Fig. S14. O-GlcNAcylation acts as a down-stream of Ca2+ influx channels. Fig. S15. Inhibition of Ca2+ influx channels and OGA knockdown impair MM cell dissemination in vivo. Fig. S16. Inhibition of Ca2+ influx channels and OGA knockdown inhibit MM spreading to BM. Fig. S17. TRPM7 depletion inhibits MM cell dissemination in vivo. Fig. S18. OGA depletion inhibits MM cell dissemination in vivo. Fig. S19. Elevated O-GlcNAcylation does not significantly associate with integrin β1 (ITGB1), β3 (ITGB3), α5 (ITGA5) and αV (ITGA [file 13046_2021_1876_MOESM2_ESM.pdf]

Supplementary Figures S1–S23

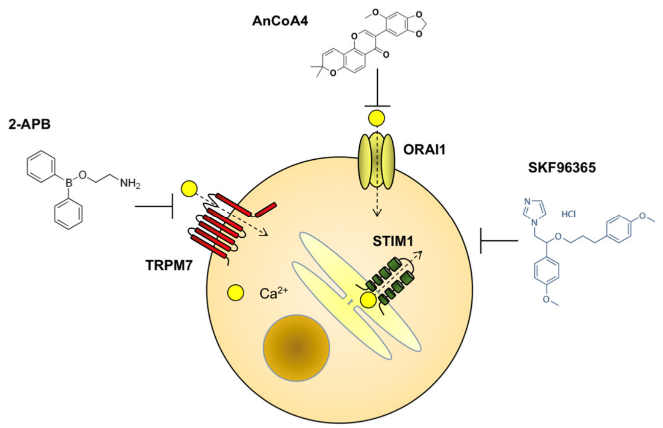

Fig. S1. Schematic representation of small molecule inhibitors (SMIs) targeting Ca^{2+} influx channels used in this study. 2-APB and SKF96365 are a nonselective modulator of Ca^{2+} influx channels used to study the impact of TRPM7 and ORAI1/STIM1 in cancers [1–3]. According to the manufacturer's datasheets, 2-APB inhibits transient receptor potential (TRP) channels in particular TRPM7 and SKF96365 inhibits STIM1, TRP-Canonical (TRPC), and voltage-gated Ca^{2+} channels. AnCoA4 is a known specific inhibitor of ORAI1 by binding to the C terminus of ORAI1 and also reducing ORAI1 binding to STIM1, causing a decreased intracellular Ca^{2+} [4].

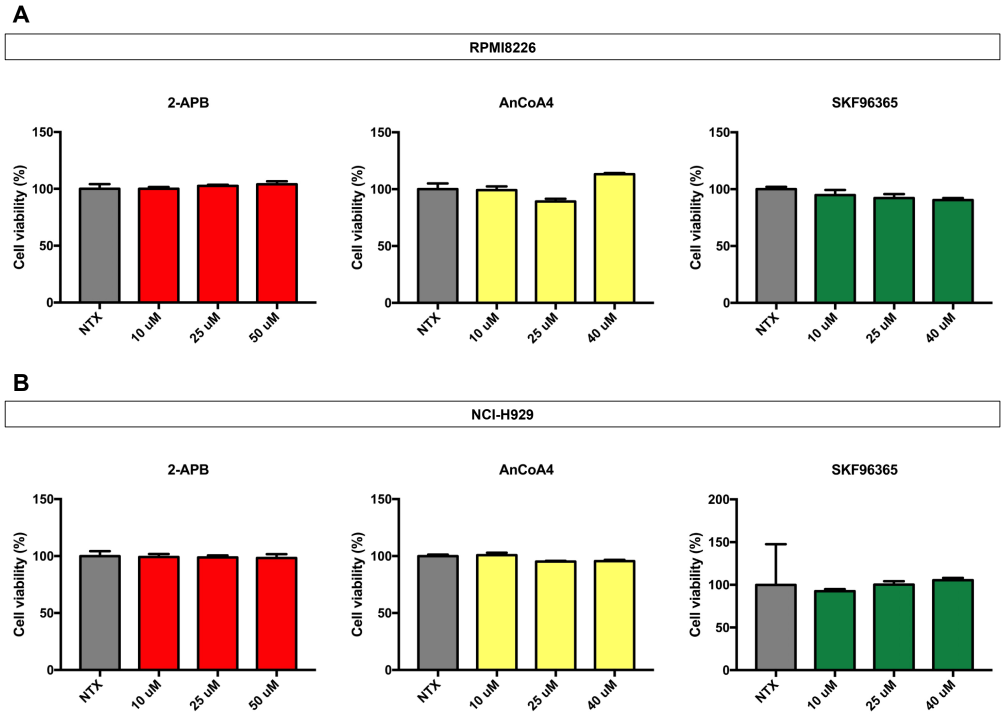

Fig. S2. Small molecule inhibition of Ca^{2+} influx channels do not affect cell viability of multiple myeloma (MM) cells. Human MM-derived RPMI8226 **(A)** and NCI-H929 **(B)** cells were treated with different doses of 2-APB (0–50 μM), AnCoA4 (0–40 μM) and SKF96365 (0–40 μM) for 48 h. The cell viability was measured by MTT assay. Data are mean ± SD (n = 4). Statistical analysis was performed using two-tailed Student's *t*-test in comparison with non-treated control (NTX).

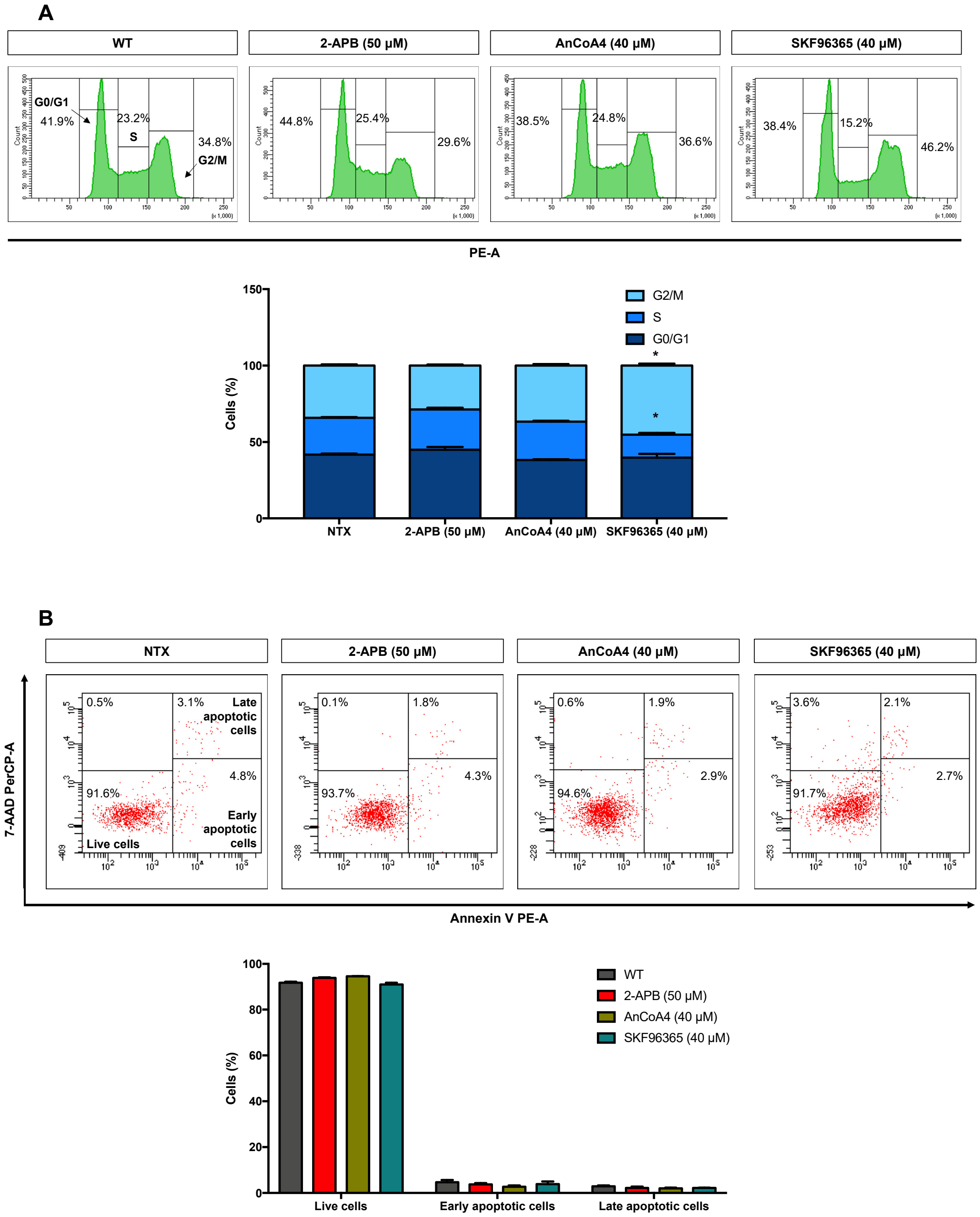

Fig. S3. Small molecule inhibition of Ca^{2+} influx channels do not affect cell cycle and apoptosis of MM cells. **A** Human MM-derived RPMI8226 cells were treated with 2-APB (50 µM), AnCoA4 (40 µM), and SKF96365 (40 µM) for 24 h and cell cycle were analyzed by flow cytometry after propidium iodide staining using CycleTEST^{TM} PLUS DNA Reagent Kit (BD Biosciences #340242). (lower) Percentage of cells in the different cell cycle phases was

calculated. **B** Apoptosis was determined by flow cytometry using annexin V-PE/ 7-AAD apoptosis assay (BD Biosciences #559763). Data are mean ± SD (n = 4). *$p < 0.05$ versus NTX; two-tailed Student's *t*-test.

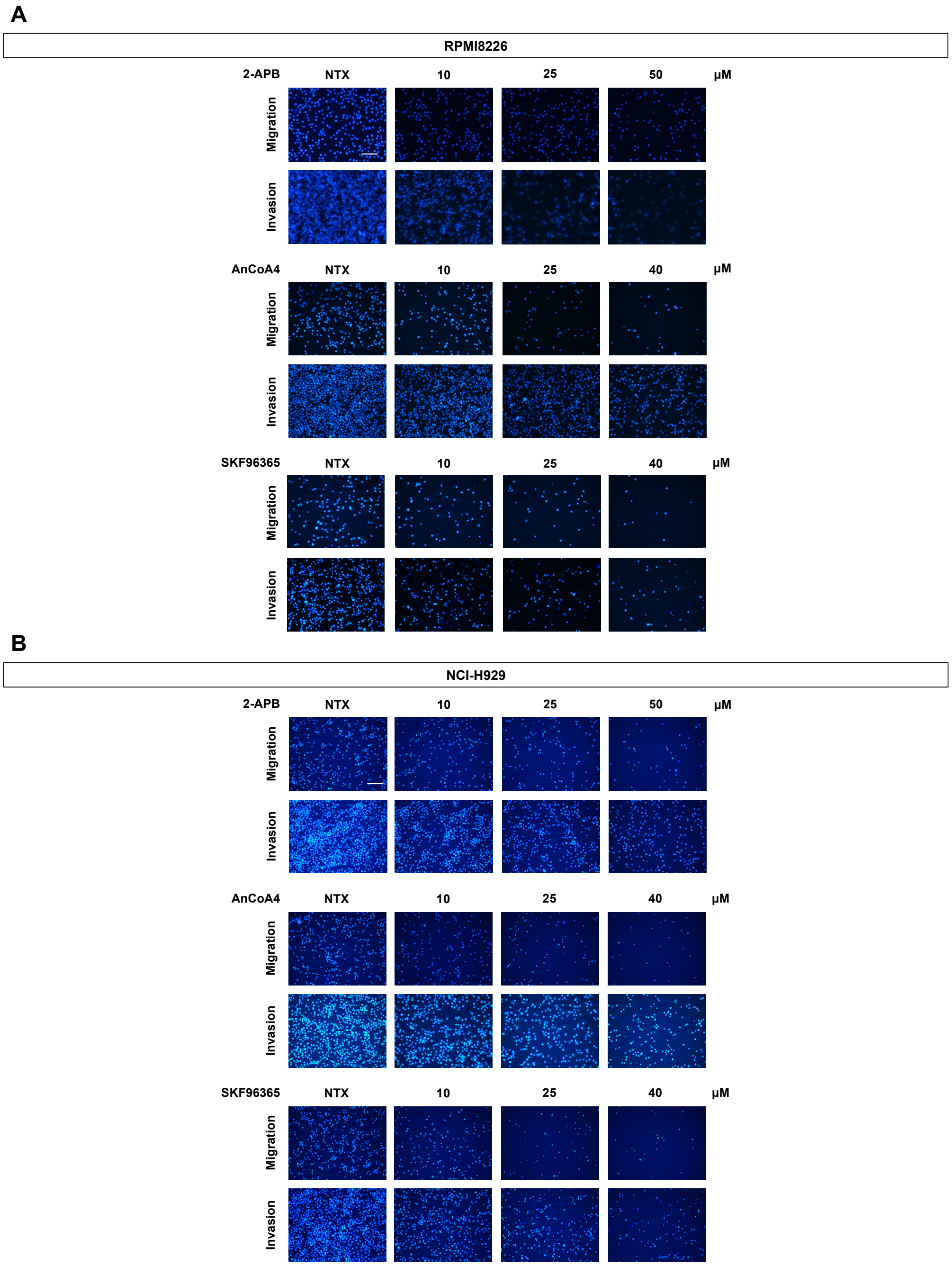

Fig. S4. Small molecule inhibition of Ca^{2+} influx channels dose-dependently inhibits MM cell motility. Human MM-derived RPMI8226 **(A)** and NCI-H929 **(B)** cells were treated with different doses of 2-APB (0–50 μM), AnCoA4 (0–40 μM) and SKF96365 (0–40 μM). Cell migration and invasion were evaluated by the Transwell assays at 24 and 48 h, respectively. Representative fields of migration and invasion cells stained with Hoechst 33342 in a 96-well plate is shown. Scale bar = 100 μm.

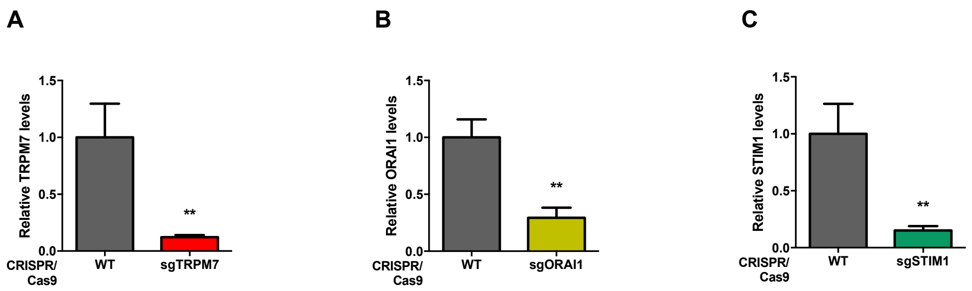

Fig. S5. Ca^{2+} influx channels sgRNA decreases the protein levels of TRPM7, ORAI1, and STIM1, respectively, in RPMI8226 cells. **A–C** Quantitative protein analysis was performed using densitometry, normalized to β-actin and relative to wild type control cells (WT), in correspond to immunoblots in **Fig. 2A–C**. Data are mean ± SD (n = 4). $**p<0.01$ versus WT; two-tailed Student's *t*-test.

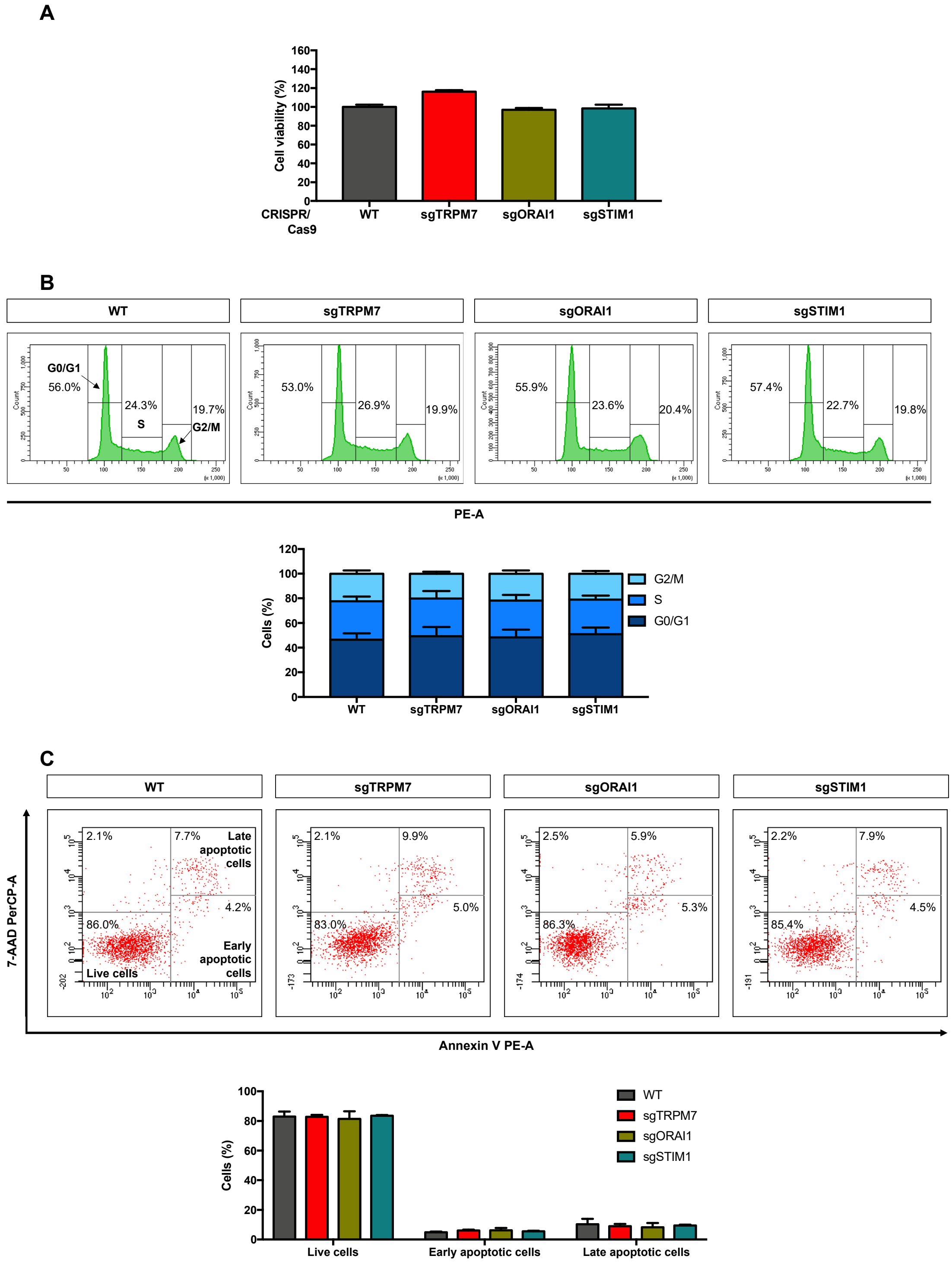

Fig. S6. Knockdown of TRPM7, ORAI1, and STIM1 do not affect cell viability, cell cycle, and apoptosis of human MM-derived RPMI8226 cells. **A** Cell viability was determined by MTT assay. **B** Cell cycle was analyzed by flow cytometry after propidium iodide staining. **C**

Apoptosis was determined by flow cytometry using annexin V-PE/ 7-AAD apoptosis assay. Data are mean ± SD (n = 4). *$p < 0.05$ versus wild type cells (WT); two-tailed Student's *t*-test.

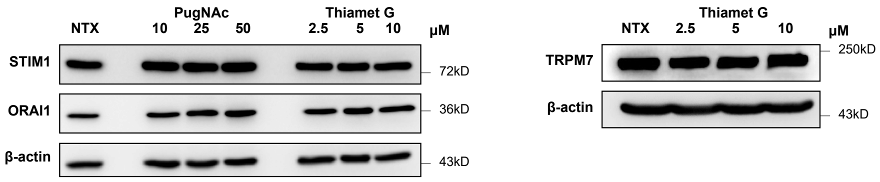

Fig. S7. Expression of Ca^{2+} influx channels are not affected by increasing levels of *O*-GlcNAc modification. Human MM-derived RPMI8226 cells were treated with various concentrations of OGA inhibitor PugNAc (0–50 μM) and thiamet G (0–10 μM) for 24 h, and STIM1, ORAI1, and TRPM7 levels were analyzed by Western blotting. β-actin was used as a loading control.

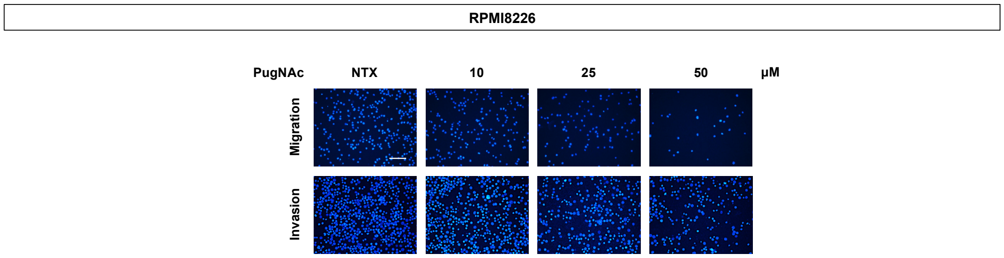

Fig. S8. Hyper-*O*-GlcNAcylation by PugNAc treatment inhibits MM cell motility. The Human MM-derived RPMI8226 was treated with different dose of PugNAc (0–50 μM). Cell migration and invasion were evaluated by the Transwell assays at 24 and 48 h, respectively. Representative fields of migration and invasion cells stained with Hoechst 33342 in a 96-well plate is shown. Scale bar = 100 μm.

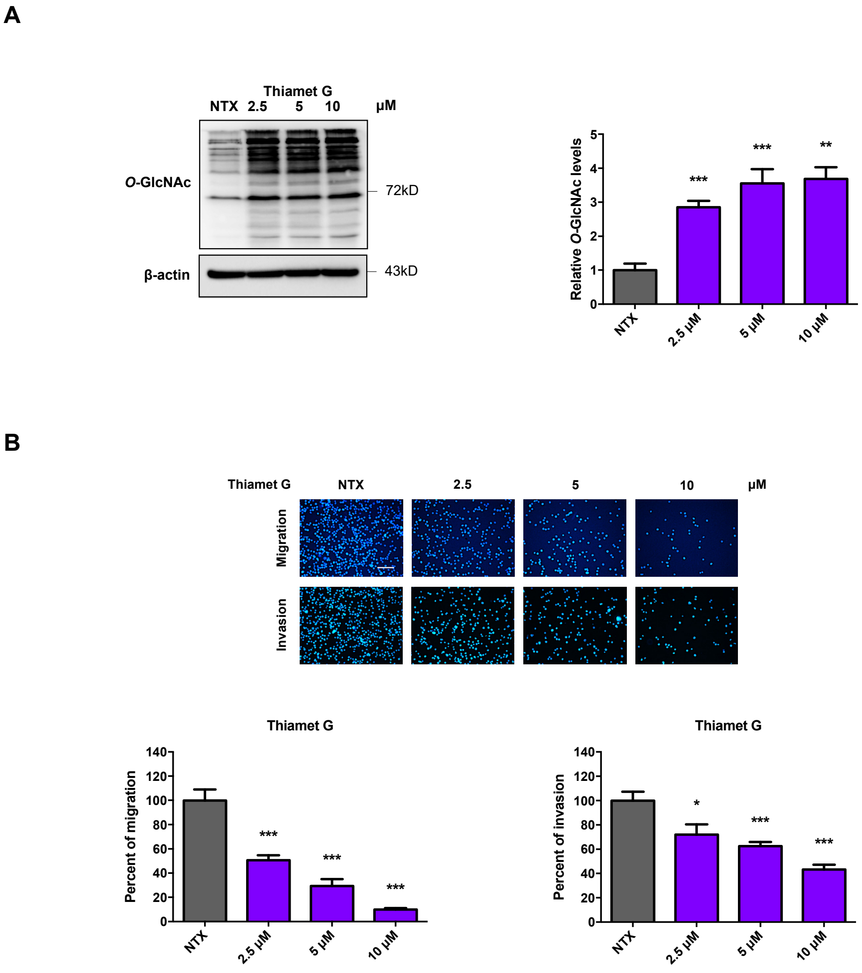

Fig. S9. Hyper-*O*-GlcNAcylation by thiamet G treatment inhibits MM cell motility. **A** Human MM-derived RPMI8226 cells were treated with various concentrations of thiamet G (0−10 μM) for 24 h and cellular *O*-GlcNAcylation was analyzed by Western blotting to confirm its induction. (right) Quantitative analysis of *O*-GlcNAcylation, normalized to β-actin and relative to the non-treated cells (NTX). Data are mean ± SD (n = 4). $**P < 0.01$, $***P < 0.001$ versus NTX; two-tailed Student's *t*-test. **B** Cell migration and invasion in response to thiamet G (0−10 μM) were evaluated by Transwell assays at 24 and 48 h, respectively, where the penetrating cells were stained with Hoechst 33342 dye. Data are mean ± SD (n = 4). $*P < 0.05$, $***P < 0.001$ versus NTX; two-tailed Student's *t*-test. Scale bar = 100 μm.

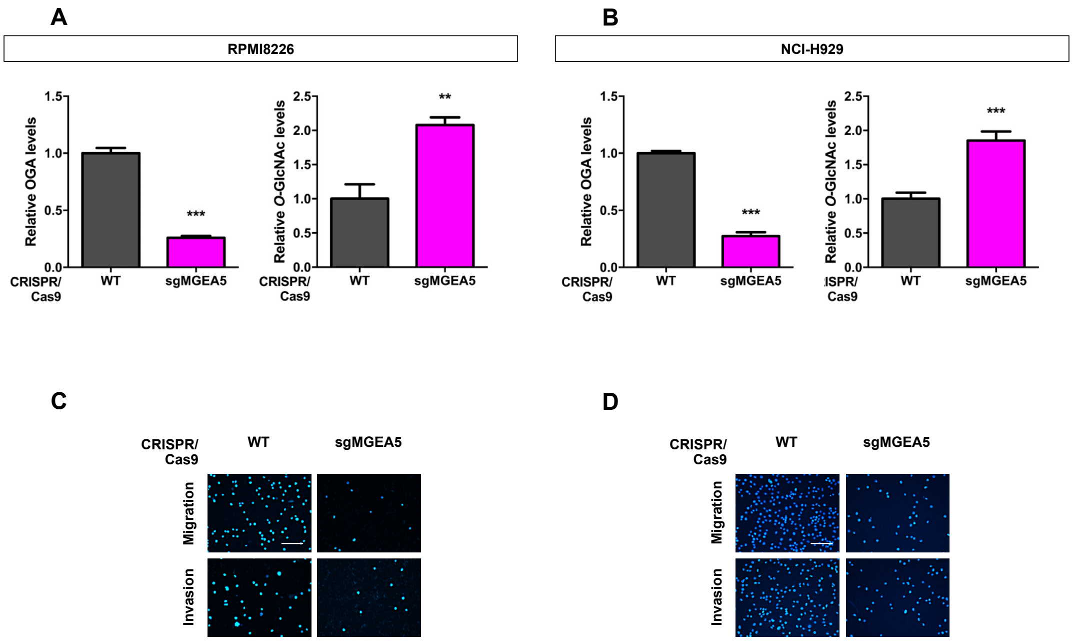

Fig. S10. Hyper-O-GlcNAcylation by sgRNA against *MGEA5* in the CRISPR/Cas9 system inhibit MM cell motility. **A, B** Quantitative protein analysis was performed using densitometry, normalized to β-actin and relative to wild type (WT) control cells, in correspond to immunoblots in **Fig. 3E and F**. Data are mean ± SD (n = 4). $^{**}p<0.01$, $^{***}p<0.001$ versus WT; two-tailed Student's *t*-test. **C, D** Cell migration and invasion were evaluated by Transwell assays at 24 and 48 h. Representative fields of migration and invasion cells stained with Hoechst 33342 in a 96-well plate is shown. Scale bar = 100 μm.

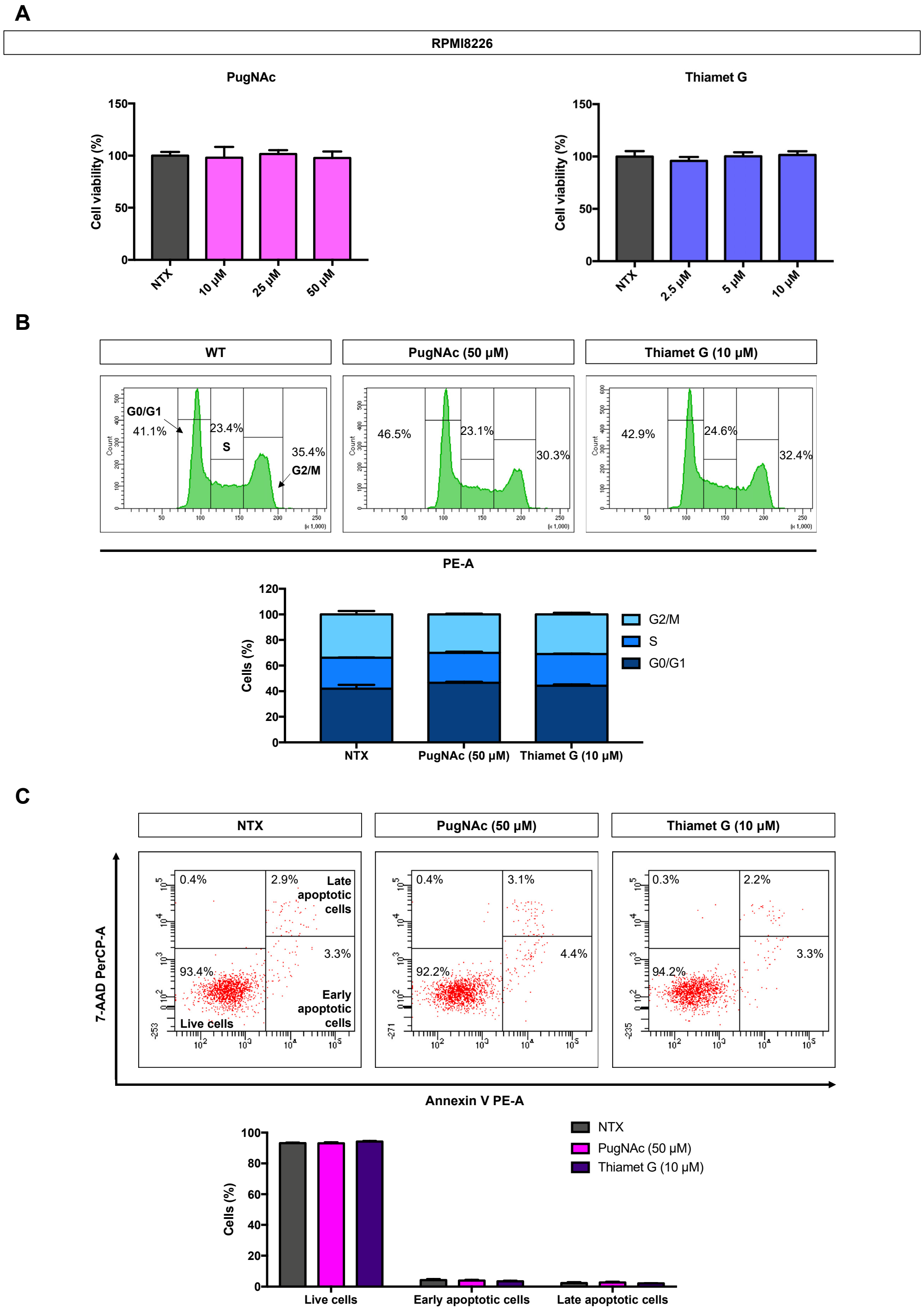

Fig. S11. Hyper-*O*-GlcNAcylation by OGA inhibition does not affect cell viability, cell cycle, and apoptosis of RPMI8226 cells. **A** Cells were treated with different doses of PugNAc (0–50 μM) and thiamet G (0–10 μM) for 48 h. The cell viability was measured by MTT assay. **B** Cell were treated with PugNAc (50 μM) and thiamet G (10 μM) for 24 h,

stained with propidium iodide, and cell cycle were determined by flow cytometry. **C** Apoptosis was determined by flow cytometry using annexin V-PE/ 7-AAD apoptosis assay. Data are mean ± SD (n = 4). Statistical analysis was performed using two-tailed Student's *t*-test in comparison with NTX.

A

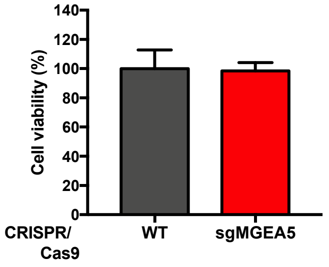

Cell viability (%)
140
120
100
80
60
40
20
0
CRISPR/ Cas9
WT
sgMGEA5

B

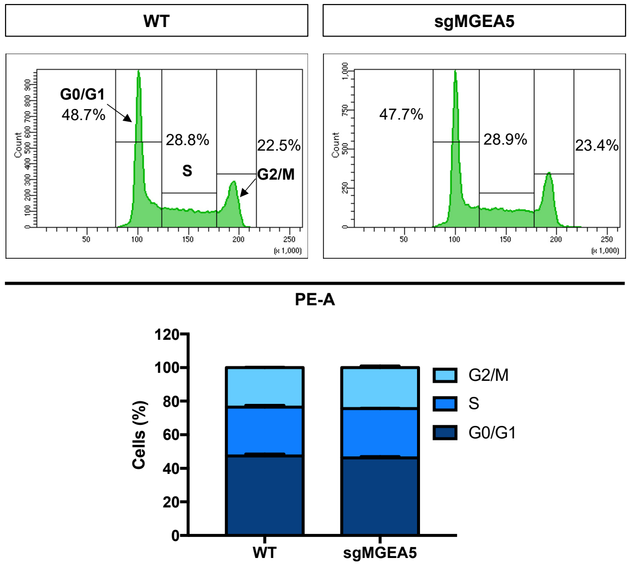

WT
sgMGEA5
G0/G1
48.7%
28.8%
S
22.5%
G2/M
47.7%
28.9%
23.4%
Count
PE-A
Cells (%)
120
100
80
60
40
20
0
G2/M
S
G0/G1
WT
sgMGEA5

C

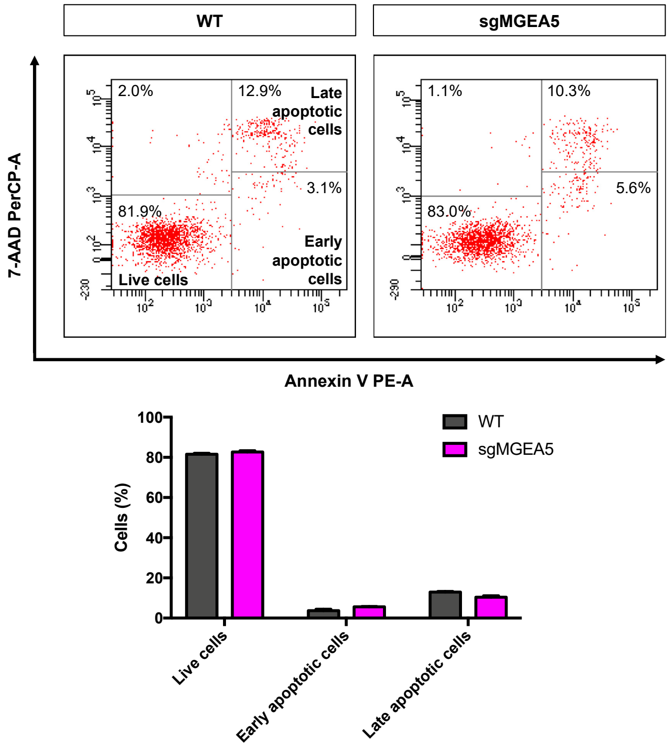

WT
sgMGEA5
2.0%
12.9%
Late apoptotic cells
3.1%
81.9%
Early apoptotic cells
Live cells
1.1%
10.3%
5.6%
83.0%
7-AAD PerCP-A
Annexin V PE-A
Cells (%)
100
80
60
40
20
0
WT
sgMGEA5
Live cells
Early apoptotic cells
Late apoptotic cells

Fig. S12. Knockdown of OGA does not affect cell viability, cell cycle, and apoptosis of human MM-derived RPMI8226 cells. **A** Cell viability was determined by MTT assay. **B** Cell cycle was analyzed by flow cytometry after propidium iodide staining. **C** Apoptosis was determined by flow cytometry using annexin V-PE/ 7-AAD apoptosis assay. Data are mean ± SD (n = 4). *$p < 0.05$ versus wild type cells (WT); two-tailed Student's *t*-test.

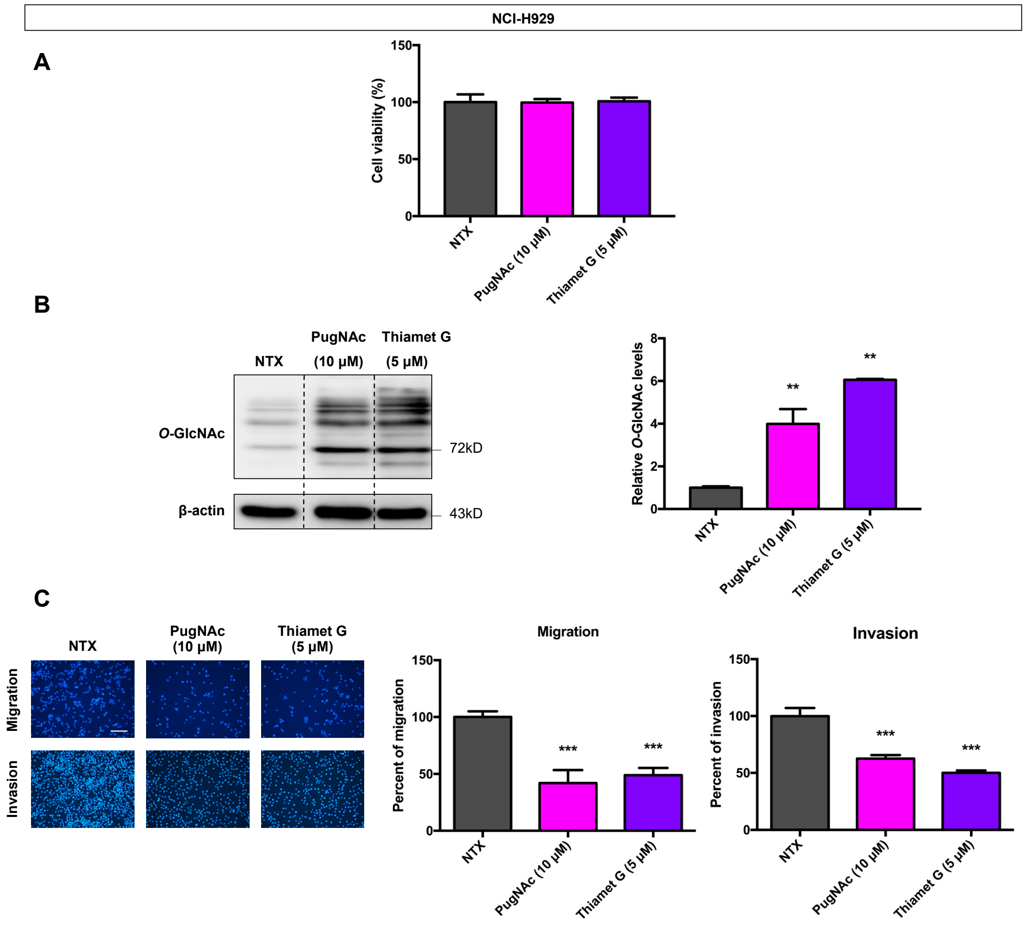

Fig. S13. Elevated *O*-GlcNAcylation similarly reduces the migration and invasion of human MM-derived NCI-H929 cells. **A** MTT assay showing that PugNAc (10 μM) and thiamet G (5 μM) treatments do not affect cell viability of NCI-H929 cells. **B** Western blot analysis of *O*-GlcNAc level in NCI-H929 cells after treatment with PugNAc (10 μM) and thiamet G (5 μM). β-actin was used as a loading control. **C.** NCI-H929 cells were treated with PugNAc

(10 μM) and thiamet G (5 μM) and cell migration and invasion were evaluated by the Transwell assays at 24 and 48 h, respectively. Representative fields of migration and invasion cells stained with Hoechst 33342 in a 96-well plate are shown. Scale bar = 100 μm. Data are mean ± SD (n = 4). **p<0.01; ***p<0.001 versus non-treated cells (NTX); two-tailed Student's t-test.

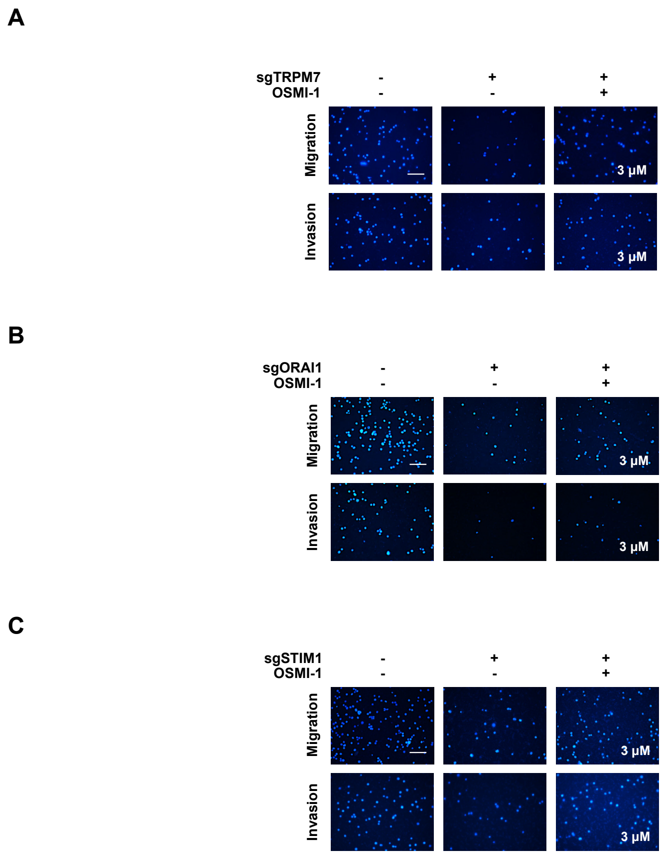

Fig. S14. *O*-GlcNAcylation acts as a down-stream of Ca^{2+} influx channels. Cells migration and invasion in sgTRPM7 **(A)**, sgORAI1 **(B)**, and sgSTIM1 **(C)** cells in presence or absence of OSMI-1 (3 μM) were evaluated by Transwell assays at 24 and 48 h, respectively. Representative fields of migration and invasion cells stained with Hoechst 33342 in a 96-well plate is shown. Scale bar = 100 μm.

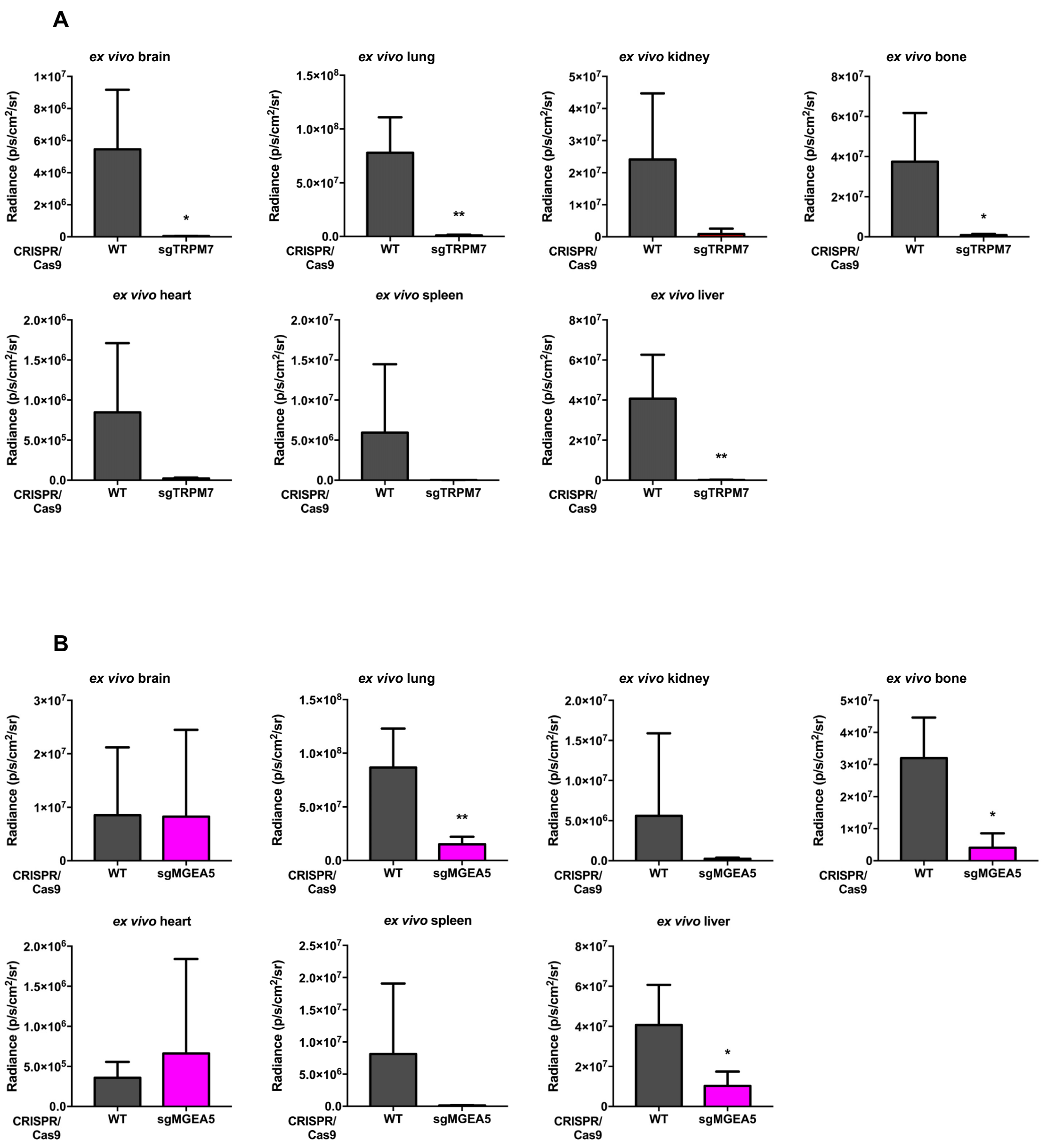

Fig. S15. Inhibition of Ca^{2+} influx channels and OGA knockdown impair MM cell dissemination *in vivo*. Quantitative *ex vivo* luciferase signal from isolated organs of mouse with luciferase-labeled sgTRPM7 **(A)** and sgMGEA5 **(B)** cells engraftment by IVIS Lumina II in Vivo Imaging system with Living Image software. Luciferase-labeled sgTRPM7 or sgMGEA5 RPMI8226 cells were injected intravenously via tail vein into NSG mice (n = 5 per group). At the end of experiment (week 4) mice were euthanized and their organs were

isolated for *ex vivo* studies according to the PerkinElmer protocol. Data are mean ± SD. *p<0.05, ***p<0.001 versus with WT group; two-tailed Student's t-test.

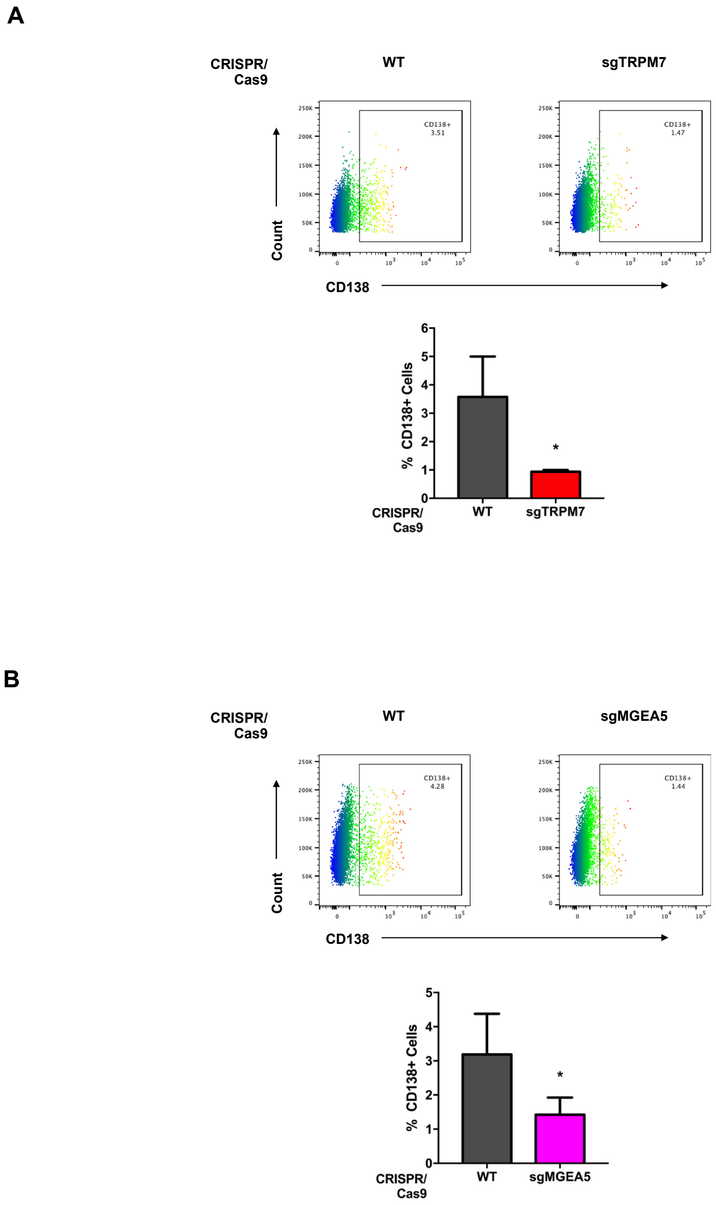

Fig. S16. Inhibition of Ca^{2+} influx channels and OGA knockdown inhibit MM spreading to BM. Cells were isolated from BM of mice engrafted with luciferase-labeled sgTRPM7 **(A)** and sgMGEA5 **(B)** cells, as described previously [5], and were subjected to flow cytometry analysis for human-$CD138^+$ cells. Representative flow cytometry plots (upper) and

quantification (lower) are shown. Data are mean ± SD. *p<0.05 versus WT group; two-tailed Student's t-test.

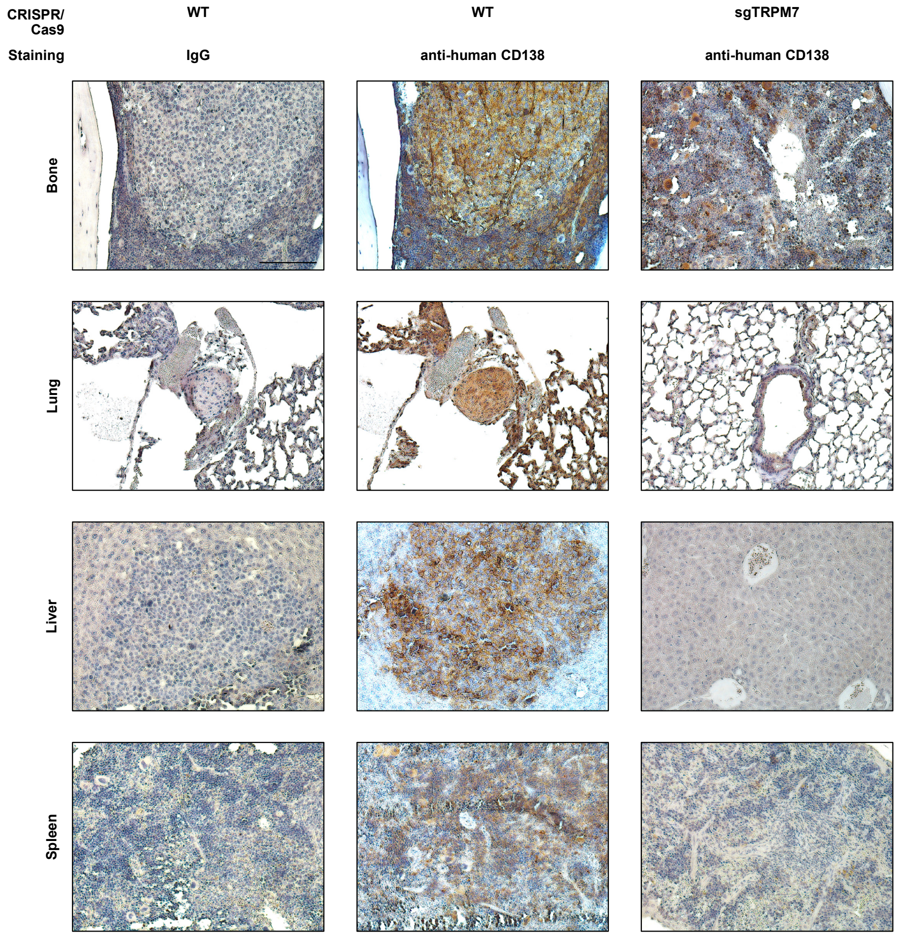

Fig. S17. TRPM7 depletion inhibits MM cell dissemination *in vivo*. Representative immunohistochemical images of organ sections, including bone, lung, liver and spleen, from MM xenograft mouse staining with anti-CD138 antibody or IgG isotype control. Scale bar = 100 μm.

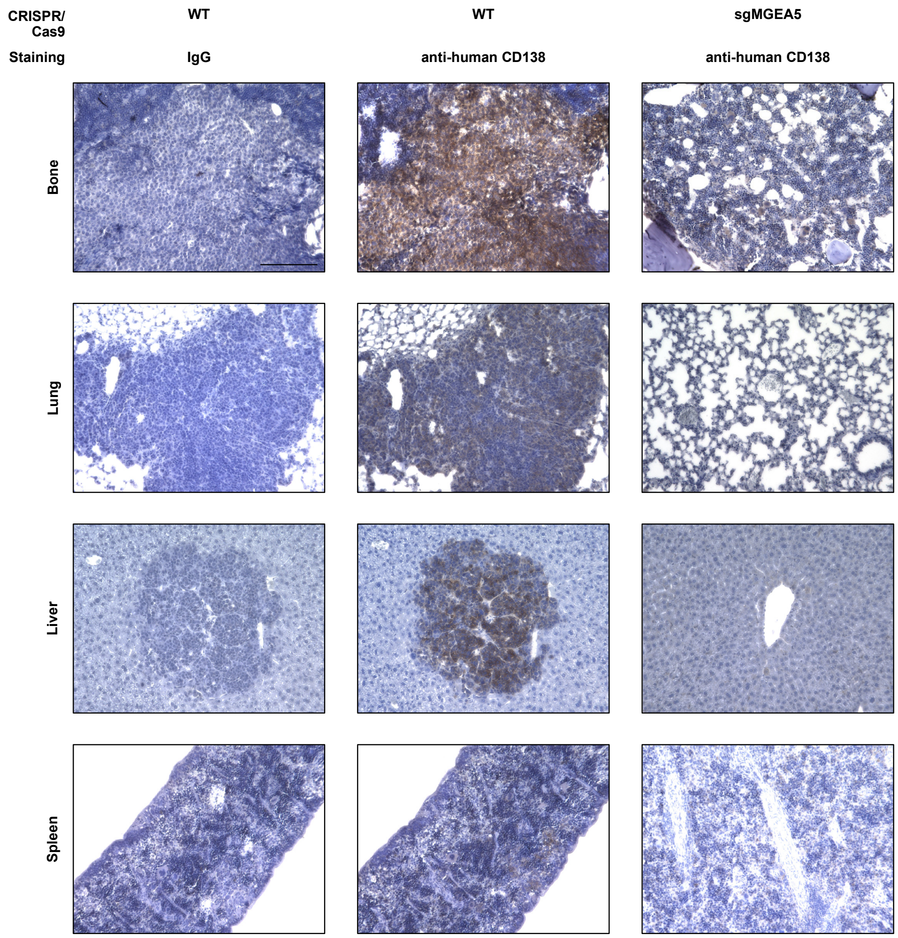

Fig. S18. OGA depletion inhibits MM cell dissemination *in vivo*. Representative immunohistochemical images of organ sections, including bone, lung, liver and spleen, from MM xenograft mouse staining with anti-CD138 antibody or IgG isotype control. Scale bar = 100 μm.

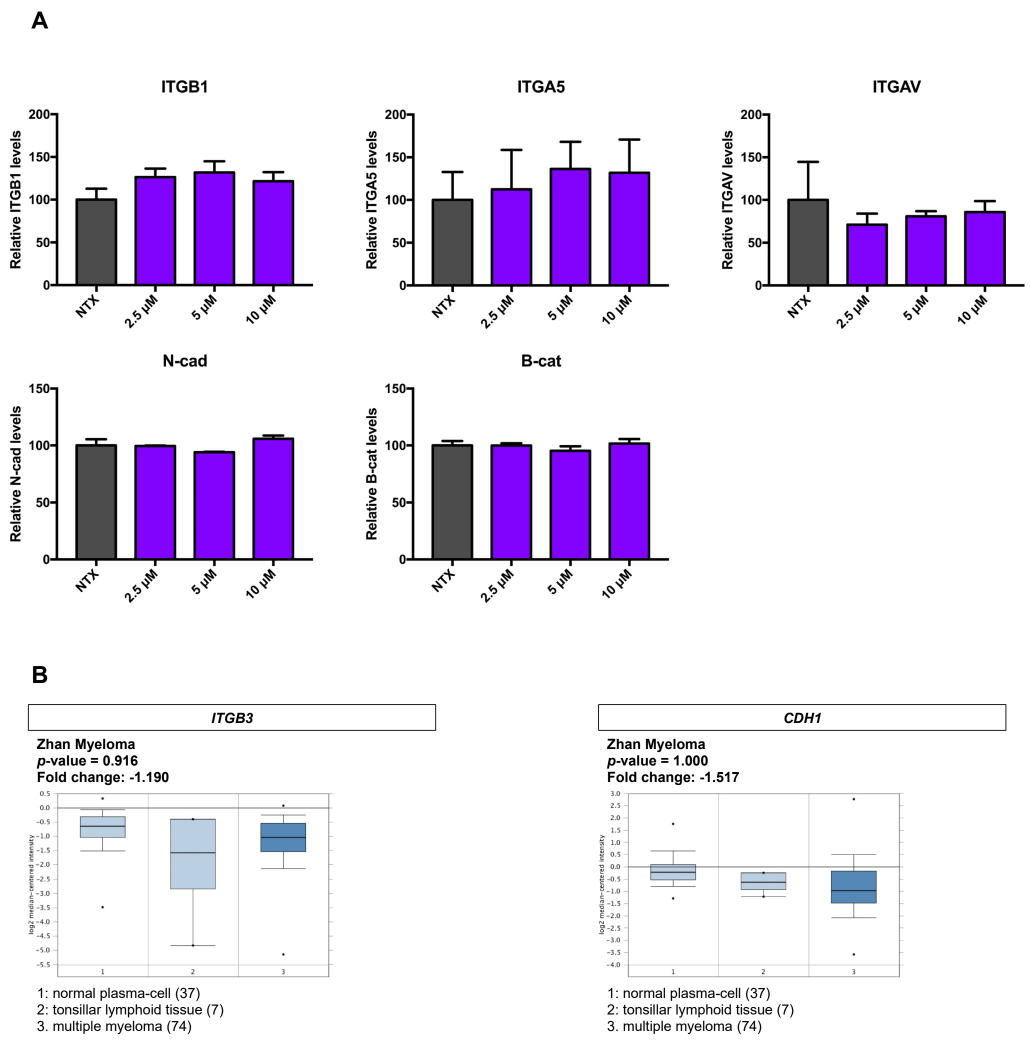

Fig. S19. Elevated *O*-GlcNAcylation does not significantly associate with integrin β1 (ITGB1), β3 (ITGB3), α5 (ITGA5) and αV (ITGAV), E-cadherin (E-cad), N-cadherin (N-cad), and β-catenin (B-cat). **A** Quantitative analysis of ITGB1, ITGB3, ITGA5, ITGAV, E-cad, N-cad, and B-cat expression of RPMI8226 cells treated with different doses of thiamet G (2.5-10 μM) in correspond to Western blot analysis in **Fig. 5a**. Data are mean ± SD (n = 4). Statistical analysis was performed using two-tailed Student's *t*-test in comparison with non-treated cells (NTX). **B** mRNA expression of *ITGB3* and *CDH1* (encoding E-cad) in clinical MM samples in comparison to normal plasma (NPC) on Oncomine™ bioinformatics database.

A

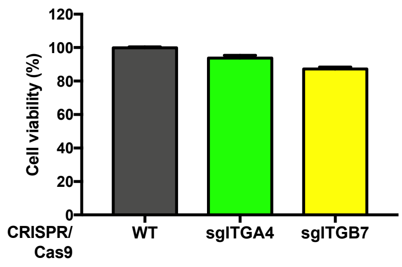
Cell viability (%)
120
100
80
60
40
20
0
CRISPR/ Cas9
WT
sgITGA4
sgITGB7

B

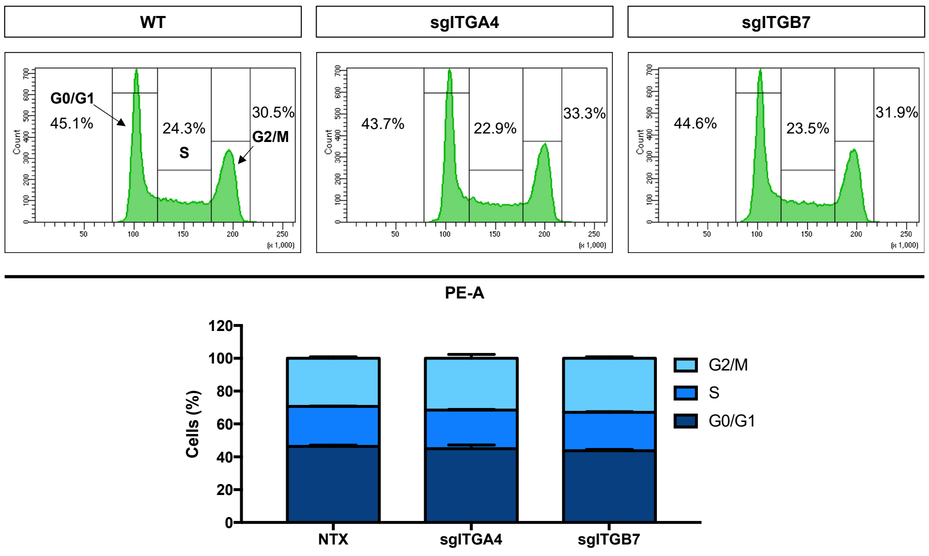
WT
sgITGA4
sgITGB7
G0/G1
45.1%
24.3%
S
30.5%
G2/M
43.7%
22.9%
33.3%
44.6%
23.5%
31.9%
PE-A
Cells (%)
G2/M
S
G0/G1
NTX
sgITGA4
sgITGB7

C

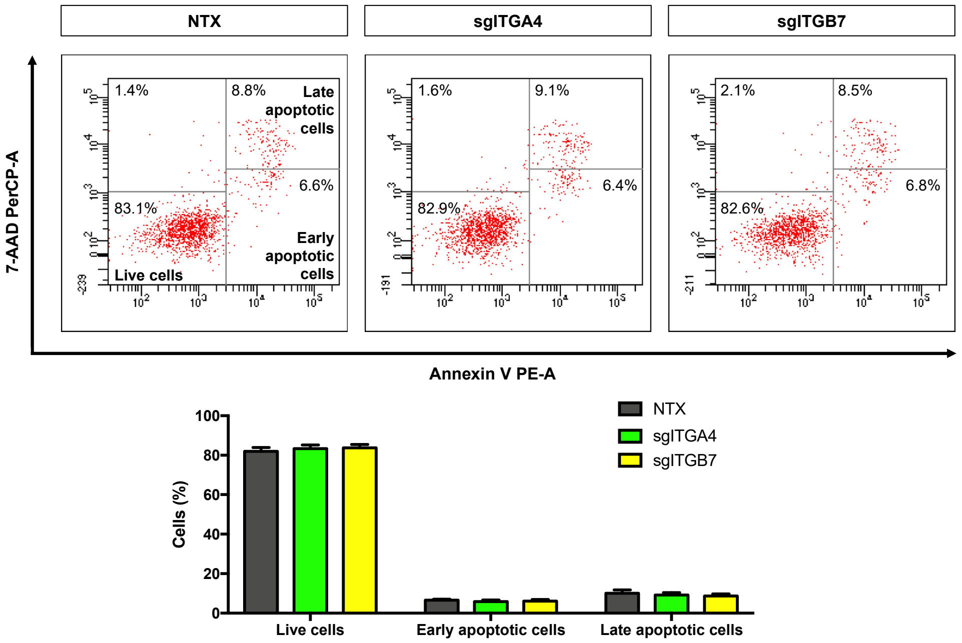
NTX
sgITGA4
sgITGB7
7-AAD PerCP-A
1.4%
8.8%
Late apoptotic cells
6.6%
83.1%
Early apoptotic cells
Live cells
1.6%
9.1%
6.4%
82.9%
2.1%
8.5%
6.8%
82.6%
Annexin V PE-A
Cells (%)
NTX
sgITGA4
sgITGB7
Live cells
Early apoptotic cells
Late apoptotic cells

Fig. S20. Knockdown of ITGA4 and ITGB7 does not affect cell viability, proliferation and apoptosis of human MM-derived RPMI8226 cells. A Cell viability was determined by MTT assays. **B** Cell cycle progression was analyzed by flow cytometry. **C** Apoptosis was analyzed by flow cytometric analysis using double labeling for annexin V-PE and 7-AAD as probes. Data are mean ± SD (n = 4). Statistical analysis was performed using two-tailed Student's *t*-test in comparison with WT.

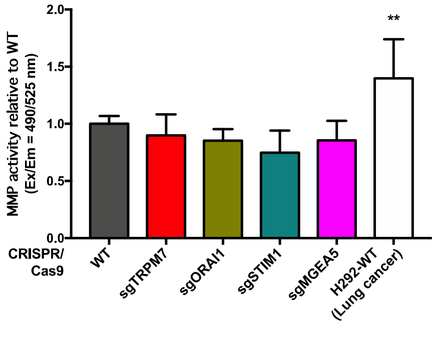

Fig. S21. Matrix metalloproteinase (MMP) activity upon an inhibition of Ca^{2+} influx channels or induction of *O*-GlcNAcylation. Cells (10^7 cells/10 mL) were cultured in serum-free RPMI1640 medium for 48 h and conditioned medium was collected and concentrated using micon Ultra-15 Centrifugal Filter Unit (Ultracel-100K membrane). Equal amounts of total protein (30 μg) were subjected to pan MMP Activity Assay Kit (Abcam, #ab112146) according to manufacturer's instructions. Data are mean ± SD (n = 4). $^{**}p < 0.01$ versus NTX; two-tailed Student's *t*-test. Human lung cancer-derived NCI-H292 cells were used as a positive control.

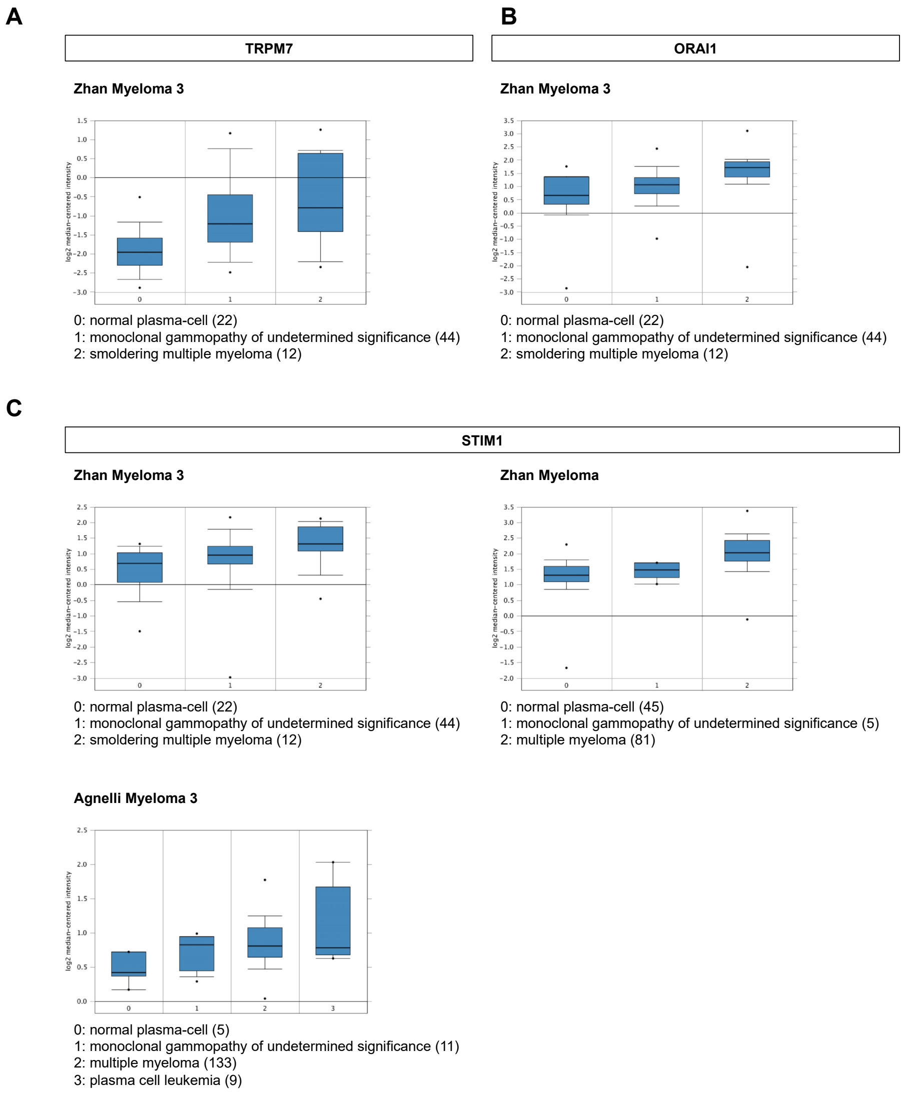

Fig. S22. Upregulation of mRNA expression of Ca^{2+} influx channels is associated with advanced MM clinical stages. **A–C** Expression of *TRPM7*, *ORAI1*, and *STIM1* in the Zhan Myeloma, Zhan Myeloma 3 and Agnelli Myeloma 3 datasets from Oncomine^{TM} bioinformatics database. MM arises from an asymptomatic pre-malignant proliferation of monoclonal plasma cells from post-germinal center of lymphoid tissue and homing to bone marrow (BM), usually known as monoclonal gammopathy of undetermined significance (MGUS) that progresses to soldering MM (SMM), symptomatic MM and finally to plasma cell leukemia.

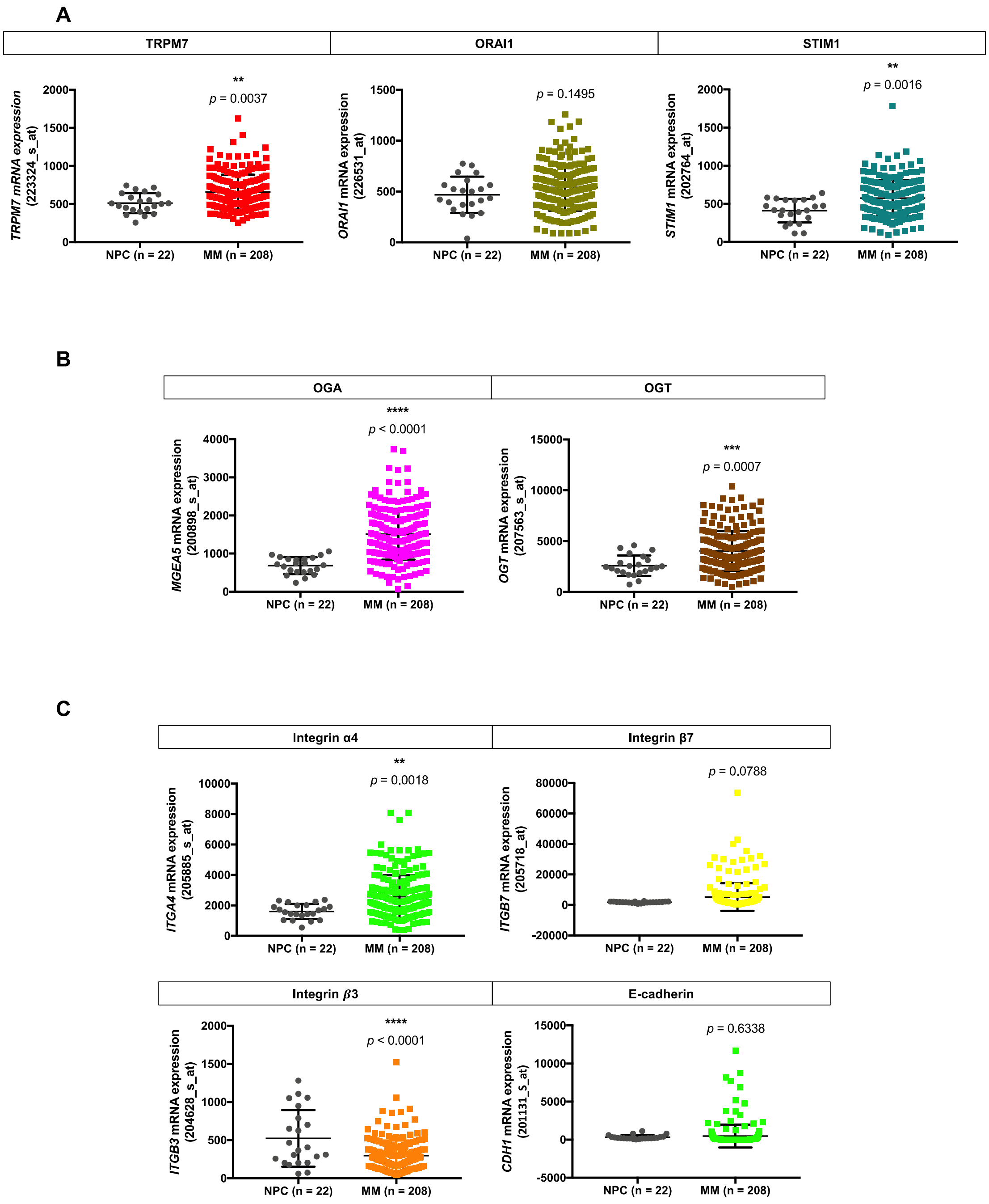

Fig. S23. mRNA expression of Ca^{2+} influx channels, *O*-GlcNAc cycling enzymes, and integrins were analyzed using microarray data available on Gene Expression Omnibus (GEO) under accession number (GSE5900 and GSE2658). NPC samples were normal plasma cells obtained from healthy donors, while MM samples from bone marrow of patients with newly diagnosed MM subsequently treated with Total Therapy 3 (pretreatment TT3). Scatter plots

of each values are shown along with the mean ± SD. $^{**}p < 0.01$, $^{***}p < 0.001$, $^{****}p < 0.0001$ versus NPC; two-tailed Student's *t*-test.

Supplementary references

1. Leng T-D, Li M-H, Shen J-F, Liu M-L, Li X-B, Sun H-W, et al. Suppression of TRPM7 inhibits proliferation, migration, and invasion of malignant human glioma cells. CNS Neurosci Ther. 2015;21:252–61.
2. Yang S, Zhang JJ, Huang XY. Orai1 and STIM1 are critical for breast tumor cell migration and metastasis. Cancer Cell. 2009;15:124–34.
3. Kim J-H, Lkhagvadorj S, Lee M-R, Hwang K-H, Chung HC, Jung JH, et al. Orai1 and STIM1 are critical for cell migration and proliferation of clear cell renal cell carcinoma. Biochem Biophys Res Commun. 2014;448:76–82.
4. Sadaghiani AM, Lee SM, Odegaard JI, Leveson-Gower DB, McPherson OM, Novick P, et al. Identification of Orai1 channel inhibitors by using minimal functional domains to screen small molecule microarrays. Chem Biol. 2014;21:1278–92.
5. Lwin ST, Edwards CM, Silbermann R. Preclinical animal models of multiple myeloma. Bonekey Rep. 2016;5:772.
